# CDK2 inhibition produces a persistent population of polyploid cancer cells

**DOI:** 10.1172/jci.insight.189901

**Published:** 2025-04-15

**Authors:** Liliya Tyutyunyk-Massey, Zibo Chen, Xiuxia Liu, Masanori Kawakami, Adam Harned, Yeap Ng, Brian Luke, Samuel C. Okpechi, Blessing Ogunlade, Yair Alfaro, Roberto Weigert, Kedar Narayan, Xi Liu, Ethan Dmitrovsky

**Affiliations:** 1 Molecular Pharmacology Program and; 2 Center for Molecular Microscopy, Cancer Research Technology Program, Frederick National Laboratory for Cancer Research, Frederick, Maryland, USA.; 3 Laboratory of Cellular and Molecular Biology, and; 4 Intravital Microscopy Core, Center for Cancer Research, National Cancer Institute, NIH, Bethesda, Maryland, USA.; 5 Advanced Biomedical Computational Science, Frederick National Laboratory for Cancer Research, Frederick, Maryland, USA.

**Keywords:** Oncology, Therapeutics, Cell cycle, Pharmacology

## Abstract

Aneuploidy, a cancer hallmark, drives chromosomal instability, drug resistance, and clinically aggressive tumors. Cyclin-dependent kinase 2 (CDK2) antagonism with independent inhibitors or CDK2 knockdown triggered anaphase catastrophe. This disrupts supernumerary centrosome clustering, causing multipolar division and apoptosis. Time-lapse fluorescence microscopy of fluorescent ubiquitination-based cell cycle indicator (FUCCI) cell cycle probes transduced into aneuploid lung cancer cells revealed distinct fates of bipolar and polyploid cells after CDK2 inhibition. Apoptosis occurred in multipolar progeny but was repressed in persistent polyploid cancer cells. RNA-Seq analyses after CDK2 inhibition of 4N versus 2N lung cancer cells were enriched for CDK1 pathway and KIF family members. The Cancer Genome Atlas (TCGA) analysis of lung cancers indicated that CDK1 and KIF family member overexpression was associated with an unfavorable survival. Intravital microscopy of transplanted lung cancer cells in mice extended findings from the in vitro to in vivo settings. CDK2 inhibition of tumor-bearing mice produced polyploid cancer cells in vivo. These cancer cells were resistant to apoptosis and proliferated despite CDK2 inhibition. In contrast, polyploid populations were rarely detected in CDK2-inhibited human alveolar epithelial cells. These findings are translationally relevant. Combined targeting of CDK2 with CDK1 or kinesin family member antagonists should eliminate polyploid cancer cells, promote apoptosis, and augment antineoplastic effects.

## Abstract 2

CDK2 inhibition disrupts centrosome clustering, triggering anaphase catastrophe in aneuploid lung cancers. Apoptosis-resistant polyploid cancer cells persist and are enriched for the CDK1 pathway and KIF families. These are antineoplastic targets.

## Introduction

Centrosome amplification is a frequent feature of hematologic and solid tumors (1). Aberrant numbers of centrosomes are linked to cancer cell aneuploidy and unfavorable patient outcomes (2–4). Normal cells tightly regulate centrosome segregation to establish 2 distinct mitotic poles, ensuring the faithful formation of a bipolar spindle (5–7). In contrast, cancer cell division engages supernumerary centrosomes that confer chromosomal missegregation. This gives rise to cellular progeny with abnormal quantity of chromosomes, known as aneuploidy. Such chromosomal imbalances can cause cell death or lead to resistance to antineoplastics and to an aggressive clinical biology (8, 9).

Centrosome amplification is associated with acquisition of a hallmark of malignancy, chromosome instability (CIN) that is often found during tumor initiation (10–12). This cancer-specific process presents an attractive target for a tumor-specific therapy that could preferentially spare normal tissues (13, 14). Disruption of centrosome clustering during mitosis follows from the inhibition of cyclin-dependent kinase 2 (CDK2) activity and is reported to promote multipolar division, which is associated with increased apoptosis (15). This proapoptotic mechanism is linked to the onset of anaphase catastrophe and to the death of progeny cells following multipolar mitosis (16–18).

Treatment with the CDK2/9 inhibitor CYC065 (Fadraciclib) having antagonism of CDK2 > CDK9 substantially increased the presence of cancer cells with multipolar mitotic spindles across diverse aneuploid cancer cells (19, 20). These effects were shown to be elicited through CDK2 and not CDK9 activities (20). In marked contrast to the actions of pharmacologic or genetically mediated repression of CDK2 activity in aneuploid cancer cells, normal human alveolar epithelial cells (HAEC) proved resistant to the growth inhibitory or proapoptotic effects of this inhibition (21). This implied that a therapeutic window existed between normal tissues and malignant aneuploid cells. This was confirmed by the presence of multipolar anaphases that were observed in syngeneic and patient-derived xenograft (PDX) models of human lung cancers (20). Independent of KRAS mutation status, substantial and statistically significant antineoplastic effects of CDK2 inhibition were observed in murine syngeneic and human PDX lung cancer models after CYC065 treatment, repressing primary and metastatic lung cancer growth in vivo (19, 20).

Despite the pronounced antitumor activity of CYC065 and other CDK2 inhibitors (15–21), a residual tumor cell population was found to persist after in vivo treatments (20). Potentially, drug resistance would occur in this population from the activation of compensatory survival mechanisms. Solid tumor cells, which are frequently aneuploid, exhibit genetic adaptations that confer resistance to treatments that cause anaphase catastrophe.

There is evidence for cellular adaptation to cell cycle inhibitors (22–25). For example, CDK2 inhibition is reported to enhance CDK4/6 activity, leading to Rb1 inactivation and subsequent cell cycle progression (26). Another adaptation is the formation of multinucleated cells, as reported in different tissues (27). This often emerges following radiation therapy treatments. This is thought to provide survival advantages and therapeutic resistance to tumors (28, 29).

This study elucidated mechanisms engaged by cancer cells to evade the death program associated with anaphase catastrophe following CDK2 inhibition. Prior work was built upon using a panel of murine and human lung cancer cell lines engineered to express 4-color fluorescent cell cycle (fluorescent ubiquitination-based cell cycle indicator [FUCCI]) probes (30, 31). Upon exposure to different pharmacologic and genetic inhibitors of CDK2, both normal HEAC and malignant lung epithelial cells were closely monitored using time-lapse live-cell microscopy.

During this observation period, multiple distinct fates of progeny cells (as compared with vehicle controls) were identified. Among the prominent fates was the frequent presence of the fusion of progeny cells after cellular division. The failure of cytokinesis, subsequent cell-cell fusion, and chromosomal missegregation promoted the presence of supernumerary centrosomes in aneuploid cancer cells. While the primary outcome of multipolar anaphase is cell death by apoptosis, the onset of cytokinesis failure leading to multinucleated cell formation was also reported here. These polyploid cancer cells persisted and proliferated despite CDK2 antagonism and contributed to the appearance of multinucleated cells that were detected in cultured lung cancer cells as well as in CYC065-treated PDX tumors transplanted into recipient mice. Intriguingly, RNA-Seq of polyploid (4N) versus diploid (2N) lung cancer cells revealed specific clusters of expressed genes that could account for the survival and persistence of polyploid cancer cells, despite ongoing CDK2 antagonism. These expressed genes were also associated with an unfavorable lung cancer survival in patients.

Our previous work showed that anaphase catastrophe is conferred by CDK2 antagonism (20). In this study, we confirm and extend that prior work using a panel of CDK2 inhibitors including several that have already entered clinical trials such CYC065 (15) and PF07104091 (Tagtociclib), a highly selective CDK2 inhibitor (32, 33). These agents were used to record the outcomes of progeny cells following the onset of multipolar mitosis.

To further uncover the fates of lung cancer cells in tumor-bearing mice after CDK2 inhibitor treatments, intravital 2-photon microscopy was used to interrogate multipolar divisions and cell fates following individual CYC065 and PF07104091 treatments. This permitted in vivo observation to be made with minimal phototoxicity of fluorescently labeled tumor cells (34). The in vivo results confirm and extend the in vitro observations to those of the in vivo lung tumor setting.

In summary, this study comprehensively explored the distinct cell fates that occur in aneuploid cancer cells that respond to CDK2 antagonism yet are not eliminated but persist in vivo despite that ongoing inhibition. The findings presented here provide a rationale for pursuit of combination CDK2-based cancer therapy. We propose that pharmacologic elimination of persistent polypoid cancer cells within lung tumors will enhance clinical efficacy of CDK2 inhibitors.

## Results

### Multipolar mitosis and multinucleated cells in lung cancer PDXs.

**Figure 1:**
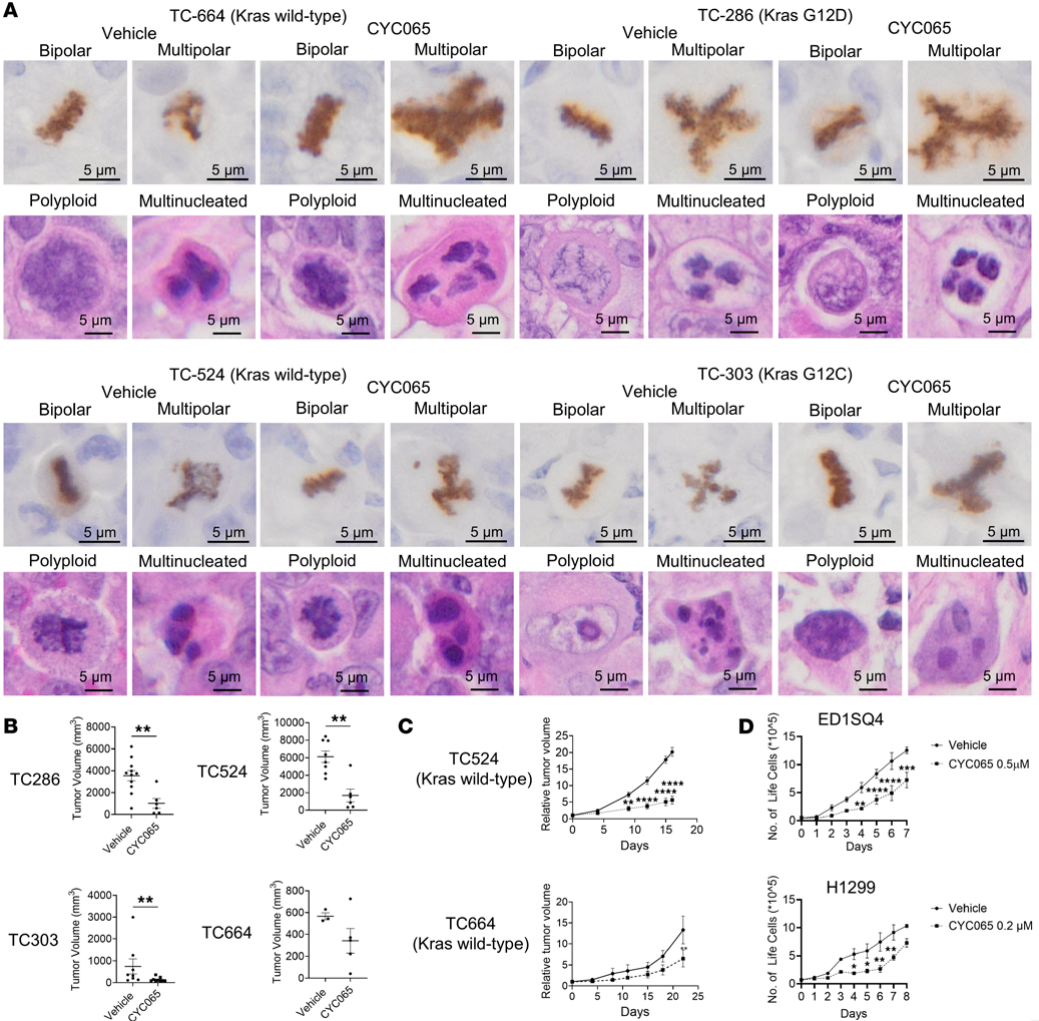
Multipolar mitosis, polyploid, and multinucleated cells were detected in human lung cancer PDX treated with vehicle or CYC065. (**A**) Representative images are shown following phospho-histone H3 and H&E staining of these different PDXs after 4 weeks of treatment with CYC065 or with vehicle as a control. KRAS WT or *KRAS* mutant mitotic cancer cells examined in these lung cancer PDXs exhibited bipolar as well as multipolar anaphases. Representative polyploid and multinucleated cancer cells are shown. (**B**) CYC065-treatment suppressed lung cancer PDX growth as compared with vehicle treatment, but persistent lung tumors were detected. (**C**) TC524 and TC664 KRAS WT lung cancer PDX models were independently treated with CYC065 versus vehicle controls and persistent in vivo tumors were detected. (**D**) ED1SQ4 and H1299 lung cancer cells rapidly grew despite CYC065 treatment after an initial phase of repression. Two-tailed Student’s *t* tests compared differences between study groups with a *P* value below 0.05 deemed statistically significant. Data are shown as mean ± SD, with the symbols indicating *P < 0.05, **P < 0.01, ***P < 0.001, and ****P < 0.0001, respectively. Scale bars: 5 μm.

The CDK2/9 inhibitor CYC065 (where CDK2 > CDK9) treatment increased multipolar mitosis in lung cancer PDXs. CYC065 treatment was selected for analyses because this agent is undergoing clinical trial testing, and its antineoplastic effects are conferred through CDK2/9 inhibition, although the death of multipolar cancer cells was largely conferred by CDK2 antagonism (20). Aberrant polyploid and multinucleated cells were independently detected in *KRAS* WT and *KRAS* mutant (G12C and G12D) lung cancers, as shown in Figure 1A. CYC065 treatment resulted in statistically significant reductions in lung tumor growth as compared with vehicle controls, as displayed in Figure 1, B and C. Notably, despite statistically significant reductions in in vivo (Figure 1C) or in vitro (Figure 1D) lung cancer growth, persistent tumor cells exist. To explore the fates of lung cancer cells that exhibit multipolar mitosis, polyploid, or multinucleated cells, it was determined whether it were possible to recapitulate in the in vitro setting of cultured lung cancer cells similar aberrant mitotic figures. Consistent with the observed antineoplastic effects of PDX lung tumors that were treated with CYC065, statistically significant growth inhibition occurred independently in human (H1299) and murine (ED1SQ4) lung cancer cell lines treated either with CYC065 at 0.2 μM (for H1299 lung cancer cells) or 0. 5μM (for ED1SQ4 lung cancer cells) drug concentrations, as in Figure 1D. The subsequent cancer cell fates of the observed progeny were monitored and scored in CYC065-teated versus vehicle-treated lung cancer cells.

### Progeny of CYC065-treated lung cancer cells.

**Figure 2:**
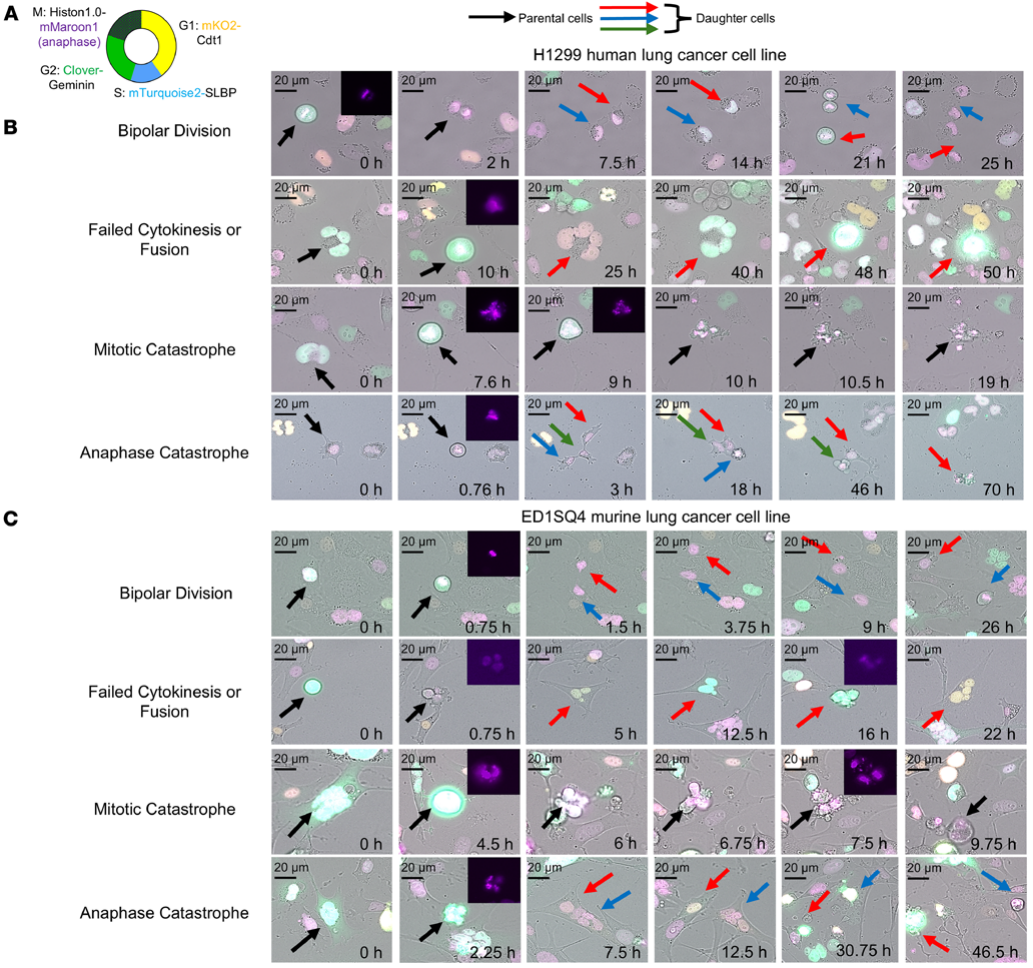
Fates of lung cancer cells after CDK2 antagonism with CYC065 treatment. The fates of the indicated lung cancer cells were monitored by live cell microscopy using fluorescent cell cycle indicator (FUCCI) probes. (**A**) The displayed diagram indicates the transfected vector that was used to probe for expression during different cell cycle phases by use of fluorescence activation in the indicated cells. This monitoring assessed the expression patterns for restricted proteins Cdt1 (yellow), SLBP (blue), and Geminin (green), corresponding to G0/G1, S, and G2/M phases, respectively. Histone 1 (purple) displayed condensed chromosomes in mitosis. (**B** and **C**) Mitotic events of human H1299 (**B**) and murine ED1SQ4 (**C**) lung cancer cells that stably expressed FUCCI vectors were treated with CYC065 or vehicle to interrogate bipolar and multipolar divisions. Indicated cells were monitored for 96 hours by time-lapse microscopy. Representative images of the fates of these cells are shown for multipolar anaphases and included failed cytokinesis, mitotic catastrophe, and anaphase catastrophe, as shown. Scale bars: 20 μm. The different displayed colored arrows indicate the respective fates of different cell progeny.

To investigate the fates as compared with vehicle control treatments of CYC065-treated lung cancer cells, live-cell microscopy assessed these cancer cell outcomes. H1299 and ED1SQ4 lung cancer cells were transduced with a FUCCI vector shown in Figure 2A. This enabled visualization of cells over an observation time period. H1299 and ED1SQ4 lung cancer cells were closely monitored during a 96-hour observation period by use of live-cell microscopy following independent CYC065 treatments at 0.2 μM and 0.5 μM drug concentrations, with results compared with that obtained after vehicle-treatment.

In contrast to bipolar mitosis, multipolar mitosis exhibited different outcomes. These were: (a) cytokinesis failure followed by fusion of progeny cells; (b) cell death during mitosis (mitotic catastrophe); or (c) death of progeny cells (anaphase catastrophe), as displayed in Figure 2, B and C. Cytokinesis failure followed by fusion of progeny cells leads to formation of multinucleated or mononucleated cellular progeny. Such fused cells can initiate a second multipolar event, as in Figure 2, B and C. The fates of A549 and H522 human lung cancer cells following CYC065 treatment appear in Supplemental Figure 1 (supplemental material available online with this article; https://doi.org/10.1172/jci.insight.189901DS1).

### Fates of CYC065-treated lung cancer cells having multipolar mitosis.

**Figure 3:**
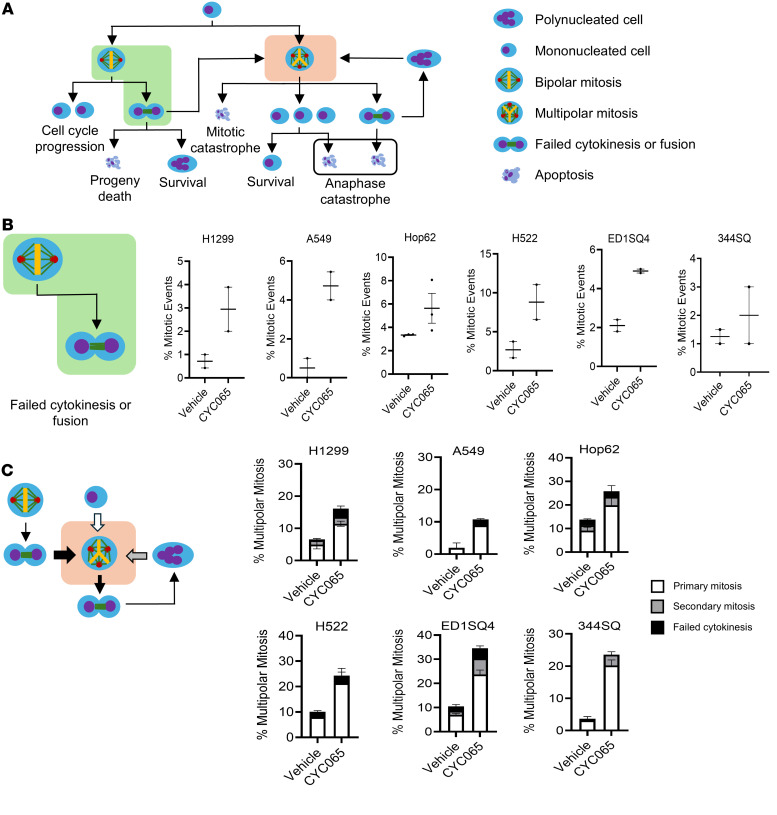
Analysis of events leading to formation of multipolar cells and those that led to multipolar anaphase. (**A**) The displayed schematic summarizes the origins of multipolar cells and of the specific cell fates that conferred multipolar mitosis. (**B**) This describes the individual fates of cytokinesis failure following bipolar divisions in human and murine lung cancer cells (human H1299, A549, Hop62, and H522 as well as murine ED1SQ4 and 344SQ lung cancer cell lines) following CYC065 treatment as compared with vehicle controls. (**C**) This schematic summarizes the quantification of events that give rise to multipolar mitotic intermediates as well as to failed cytokinesis following bipolar mitosis formation and the onset of supernumerary centrosomes in mononucleated and multinucleated cells prior to the onset of cell division. Two-tailed Student’s *t* tests compared differences between study groups with a *P* value below 0.05 deemed statistically significant. Data are shown as mean ± SD.

Live-cell imaging of cells treated with CYC065 as compared with vehicle controls revealed that lung cancer cells that exhibited multipolar mitosis arose from 1 of these 3 major pathways: failed cytokinesis followed by fusion of progeny cells, lung cancer cells with supernumerary centrosomes, and cancer cells that exhibited multinucleated states. The predominant fates of the progeny of lung cancer cells with multipolar mitosis after CDK2 inhibition were cell death during mitosis (mitotic catastrophe), a surviving population, cell death outside of mitosis, or failed cytokinesis followed by fusion of progeny cells leading to a second multipolar mitotic event. The origins and fates of lung cancer cells treated with CYC065 that exhibited multipolar mitosis are summarized in Figure 3A.

To elucidate the proportion of lung cancer cells that have distinct fates following CYC065 treatment, a panel of human and murine lung cancer cell lines was independently examined. Each cell line was independently exposed to CYC065 at drug concentrations of 0.2 μM and 0.5 μM for 96 hours, and then the cancer cell progeny was scored based on FUCCI signals using live-cell microscopy. Increased cell fusion occurred following CYC065 treatment in all these studied lung cancer cell lines, as presented in Figure 3B. CYC065 treatment promoted the onset of supernumerary centrosome formation in lung cancer cellular progeny that exhibited multipolar mitosis in the primary mitotic event. Cytokinesis failure and cell fusion following multipolar mitosis led to the presence of multinucleated cells that formed during the second mitotic event, causing multipolar mitosis as seen in Figure 3C.

As multipolar mitosis conferred by CYC065 treatment typically occurred at subtherapeutic drug concentrations (20), the fates of lung cancer cells exposed to higher concentrations of CYC065 such as at the 1 μM drug dosages were studied. The lung cancer progeny of these studies exhibited G2 arrest that was followed by cell death, as shown in Supplemental Figure 2. Mitotic events were not typically observed at this higher concentration of CYC065 treatment.

### Lung cancer cell fates after multipolar mitosis.

**Figure 4:**
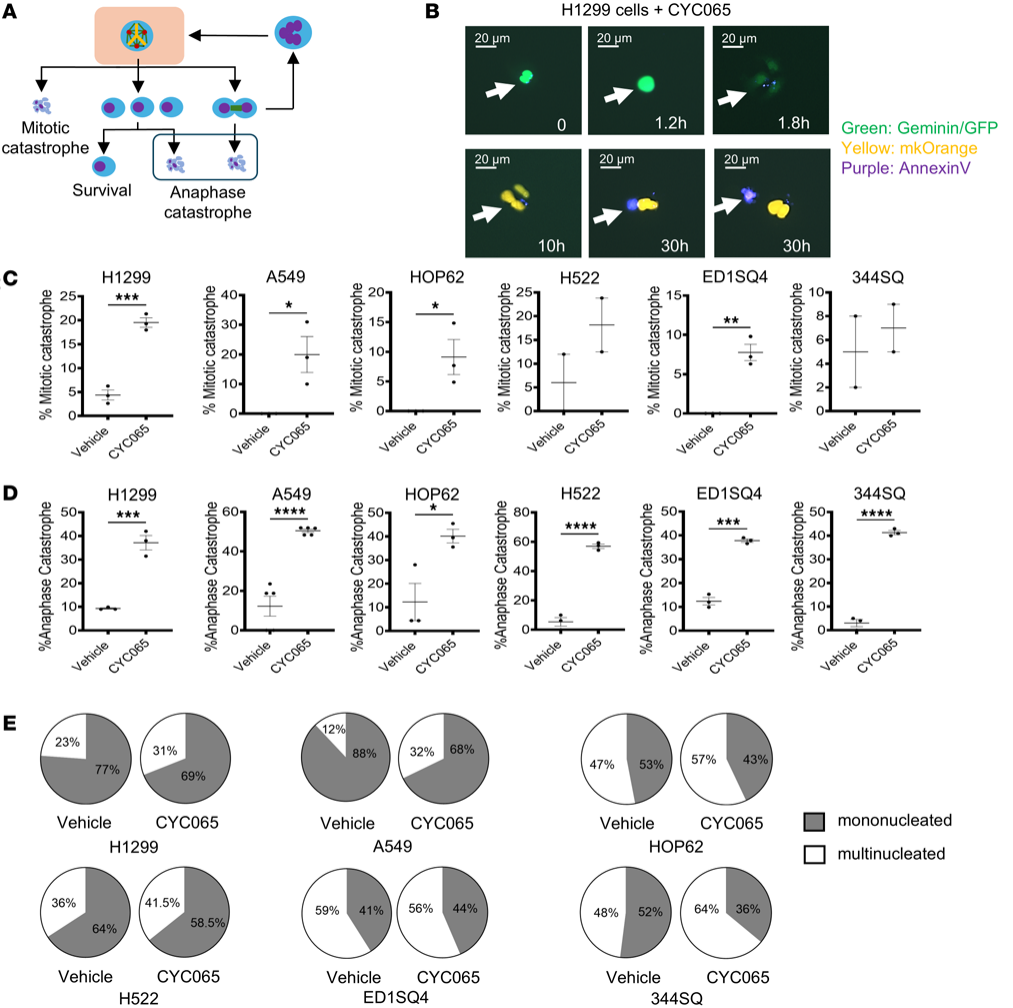
Analysis of cell fates following the independent onset of multipolar mitosis in human and murine lung cancer cells. (**A**) This schematic summarizes the cancer cell fates that arise after the appearance of multipolar mitosis in CYC065-treated cancer cells (human H1299, A549, Hop62, and H522 as well as murine ED1SQ4 and 344SQ lung cancer cell lines) that led progeny to survive, exhibit mitotic catastrophe, elicit anaphase catastrophe, or develop failed cytokinesis followed by multinucleated cell formation. (**B**) Representative images are displayed for apoptotic human H1299 lung cancer cells treated with CYC065 (0.2 μM). Yellow fluorescence (mKO) and green fluorescence (Geminin) measured the presence of G0/G1and G2/M cell cycle phases, respectively. The purple stain (annexin V) indicated the onset of apoptosis. (**C**) Mitotic catastrophe as an outcome of multipolar mitosis is caused by CYC065 treatment relative to vehicle treatment. (**D**) Anaphase catastrophe occurred after multipolar mitosis after CYC065 versus vehicle treatments. (**E**) The accumulation of multinucleated cells was caused by CYC065 treatment as compared with vehicle treatment. Two-tailed Student’s *t* tests compared differences between study groups, with a *P* value below 0.05 deemed statistically significant. Data are shown as mean ± SD, with the symbols indicating *P < 0.05, **P < 0.01, ***P < 0.001, and ****P < 0.0001, respectively. Scale bars: 20 μm. Each arrow follows the fates of individual cancer cells over time.

Anaphase catastrophe is the death of progeny cancer cells after multipolar mitosis (18). This contrasts with the onset of mitotic catastrophe that occurs during the first mitosis after cancer cell division (35). This study built on this prior work by elucidating the proportion of cancer cells that elicit anaphase catastrophe relative to mitotic catastrophe during multipolar mitosis after CDK2 inhibition. Live-cell microscopy visualized cellular fates of multipolar mitotic events, as shown in Figure 4A. The majority of the displayed H1299 and A549 lung cancer cells exhibited a marked apoptotic response after either CYC065 treatment at the 0.2 μM or 0.5 μM drug concentrations, as evidenced by the prominence of treated cancer cells that exhibited annexin V staining, as displayed in Figure 4B and Supplemental Figure 3, A and B.

CYC065 treatment caused a statistically significant increase in the number of cancer cells exhibiting mitotic catastrophe as compared with vehicle treatments as shown in Figure 4C. CYC065 treatment statistically significantly augmented the onset of anaphase catastrophe in progeny cancer cells that exhibited apoptotic death, as in Figure 4D. There were 2 major pathways observed that gave rise to anaphase catastrophe following CDK2 antagonism. These were progeny cells that exhibited multipolar mitosis or failed cytokinesis. CYC065 treatment also increased the proportion of multinucleated progeny after multipolar mitosis. This cancer cell progeny underwent a second multipolar mitotic event, as seen in Figure 4E.

### Multinucleated cancer cells.

**Figure 5:**
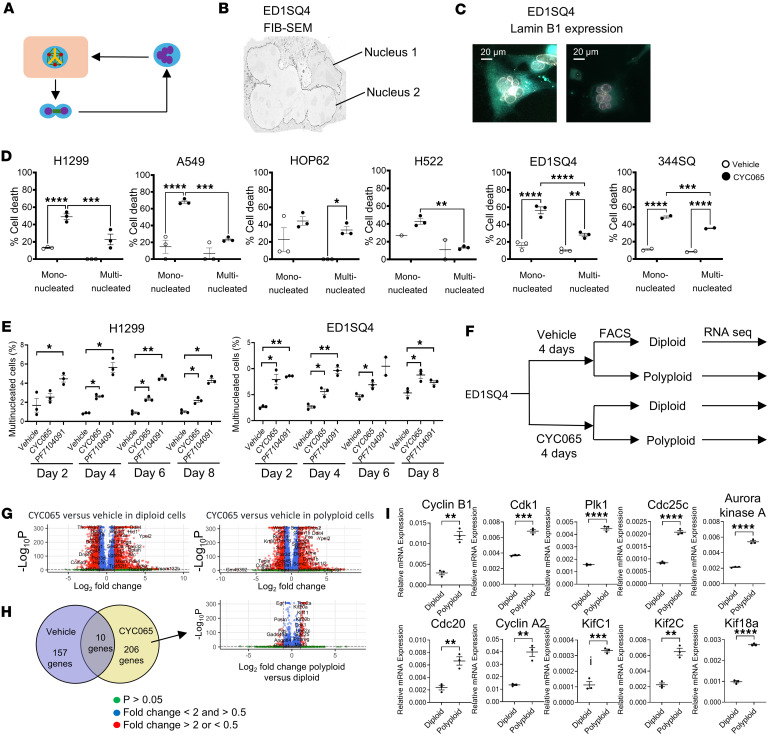
The characterization of multinucleated cells after CDK2 inhibition. (**A**) This schematic summarizes the cycling cancer cell fates: multipolar mitosis followed by cytokinesis failure, which gives rise to multinucleated cancer cells that can enter the next multipolar mitosis. (**B**) A representative image of a multinucleated cancer cell is shown by FIB-SEM. (**C**) Immunofluorescence staining of Lamin B1 for nuclear membrane confirmed the multinucleated phenotype. (**D**) Apoptotic cell death was enhanced by CYC065-treated mononucleated and multinucleated cancer cells (human H1299, A549, Hop62, and H522 as well as murine ED1SQ4 and 344SQ lung cancer cell lines), with multinucleated cancer cells having a statistically significant lower apoptosis rate than did mononucleated cells. (**E**) The ratios of multinucleated cancer cells/total cells remained stable during 8 days of CYC065 treatments in H1299 and ED1SQ4 cells. (**F**) Diploid (2N) and polyploid (4N) ED1SQ4 lung cancer cells were isolated by FACS for RNA-Seq analysis following vehicle or CYC065 treatment. (**G**) Volcano plots of differentially expressed genes caused by CYC065 as compared with vehicle treatment were displayed for diploid and polyploid cancer cells. Red dots indicate expressed genes with fold change > 2 or < 0.5 and with *P* < 0.05. Blue dots indicate expressed genes with fold change < 2 and > 0.5 and with *P* < 0.05. Green dots indicate expressed genes with *P* > 0.05. (**H**) Volcano plot and Venn diagram of the differentially expressed genes in polyploid versus diploid cancer cells augmented by CYC065 treatments are displayed. Differential gene expression analysis (**G** and **H**) was performed using a negative binomial model and Wald test with Benjamini-Hochberg correction to calculate adjusted *P* values. (**I**) Real-time PCR assays validated the representative differentially expressed genes (*Ccnb1*, *Ccna2*, *Cdk1*, *Plk1*, *Aurka*, *Cdc25c*, *Cdc20*, *Kif18a*, *Kif2c*, and *Kifc1*) increased in polyploid versus diploid cancer cells by CYC065 treatment. Two-tailed Student’s *t* tests compared differences between study groups in **D**, **E**, and **I** with a Bonferroni correction of *P* value below 0.05 deemed statistically significant. Data are shown as mean ± SD, with the symbols indicating *P < 0.05, **P < 0.01, ***P < 0.001, and ****P < 0.0001, respectively. Scale bars: 20 μm.

CYC065 treatment increased the proportion of cells having multipolar mitosis. This rendered some of these cancer cells to exhibit failed cytokinesis, which gives rise to multinucleated cells, as seen in Figure 5A. Focused-ion-beam scanning electron microscopy (FIB-SEM) was used to analyze the ultrastructure of these multinucleated cells. This confirmed that failed cytokinesis or fusion of progeny cells after CDK2 inhibition augmented multipolar division, leading to the formation of multinucleated progeny in Figure 5B. These findings were confirmed and extended by use of FIB-SEM analysis of the CYC065-treated human H1299 lung cancer cell line, as seen in Supplemental Figure 3C. Immunofluorescence staining of Lamin B1 independently confirmed that the displayed cancer cells were multinucleated, as shown in Figure 5C.

Notably, the multinucleated cancer cell progeny after multipolar mitotic events had a statistically significantly lower rate of apoptosis than did mononucleated cells, as displayed in Figure 5D. However, the ratio of multinucleated cells/mononucleated cells remained stable during 8 days of CYC065 or PF7104091 treatment, as in Figure 5E. This indicated that this population of progeny cancer cells persisted despite continued CDK2 inhibition.

FACS based on chromosomal content was used to isolate independently 4N and 2N cancer cells following 72 hours of vehicle or CYC065 treatments. These respective populations were harvested and used for RNA-Seq analysis, as in Figure 5F. Differentially increased or repressed genes that were most prominently affected by CYC065 treatment versus vehicle treatment were identified in Figure 5G. This study sought to elucidate the gene expression profiles that discriminated between polyploid and diploid cancer cells following CDK2 inhibition, as shown in Figure 5H and Table 1. Real-time PCR assays validated upregulated expressed genes such as *Ccnb1*, *Ccna2*, *Cdk1*, *Plk1*, *Aurka*, *Cdc25c*, Cdc20, *Kif18A*, *Kif2c*, and *Kifc1*, as shown in Figure 5I and Supplemental Figure 4.

Intriguingly, the expressed genes of CDK1 pathway and Kinesin family members (KIF) were upregulated or downregulated in polyploid cells as compared with diploid cancer cells treated with CYC065 as shown in Table 1 and Table 2. It was reported that prolonged CDK2 inhibition increased cancer cell reliance on the CDK1 pathway, while rapid adaptation depended on expression of the CDK4/6 pathway (26). Among 13 KIF family members with augmented expression within polyploid versus diploid cancer cells after CYC065 treatment, *Kifc1* (36, 37), *Kif2c* (38), *Kif22* (39, 40), *Kif18a* (41), and *Kif11* (42, 43) were altered in their expression. Each of these expressed genes are involved in CDK1 pathway regulation or in centrosome clustering of cancer cells having supernumerary centrosomes.

**Figure 6:**
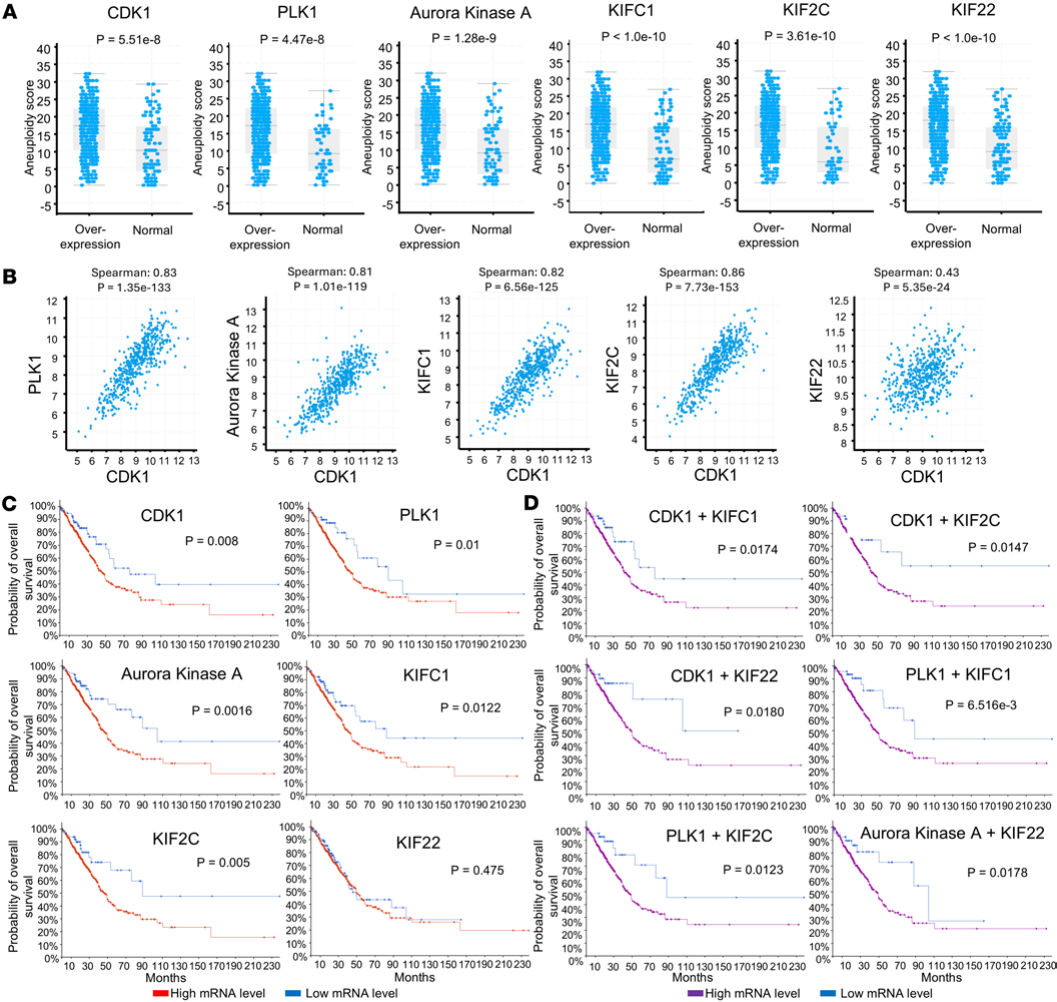
Overall survival of patients with lung cancer. Overall survival of patients with lung cancer having these differentially expressed genes (CDK1, PLK1, AURKA, KIFC1, KIF2C and KIF22) in polyploid versus diploid cancer cells after CYC065 but not vehicle-treatments within the displayed lung adenocarcinoma cohort (566 patients) as part of the PanCancer Atlas in The Cancer Genome Atlas (TCGA). (**A**) Overexpression of CDK1, PLK1, AURKA, KIFC1, KIF2C or KIF22 was significantly associated with a high aneuploidy score. The Wilcoxon test was used to compare aneuploidy scores of the examined patients with *P* values below 0.05 deemed statistically significant. (**B**) CDK1 expression was statistically significantly associated with the expression profiles of PLK1, AURKA, KIFC1, and KIF2C but weakly for KIF22. The Spearman’s rank correlation coefficient was used to test the indicated associations, with *P* values below 0.05 deemed statistically significant. (**C**) Overexpression profiles of CDK1, PLK1, AURKA, KIFC1, and KIF2C indicate an unfavorable overall survival of patients with lung cancer. KIF22 expression in lung cancer did not affect overall survival. (**D**) The combined overexpression profiles of CDK1 + KIFC1, CDK1 + KIF2C, CDK1 + KIF22, PLK1 + KIFC1, PLK1 + KIF2C, and AURKA + KIF22 were associated with an unfavorable overall survival in the examined patients with lung cancer. Survival analysis for **B** and **D** was performed using the Kaplan-Meier method using the log-rank test, with a *P* value below 0.05 deemed statistically significant.

A bioinformatics study of kinesin family members in lung adenocarcinomas highlighted functional interactions between these datasets (44). To explore the clinical relevance of these regulated genes, RNA-Seq data from the lung adenocarcinoma cohort (566 patients) in the PanCancer Atlas within The Cancer Genome Atlas (TCGA) (45) were used for bioinformatics analysis of these genes. Those genes that were augmented in expression for the CDK1 pathway and KIF family members appear in Figure 6 and Supplemental Figure 5. These genes included *CDK1*, *PLK1*, *AURKA*, *KIFC1*, *KIF2C*, and *KIF22*. Each of them was associated with a higher aneuploidy score of the indicated lung cancers, as presented in Figure 6A and Supplemental Figure 5A. The expression profile of the upregulated CDK1 pathway members showed a statistically significant association with the expression of those upregulated *KIF* genes except for *KIF22* that had a weak association, as determined in Figure 6B and Supplemental Figure 5B.

Survival analysis revealed that overexpression of *CDK1*, *PLK1*, *AURKA*, *KIFC1*, and *KIF2C* was associated with a statistically significant unfavorable overall survival in patients with lung cancer. In contrast, *KIF22* expression in lung cancers did not predict this unfavorable survival outcome, as shown in Figure 6C. Supplemental Figure 5C shows lung cancer survival outcomes for other members of the *CDK1* and *KIF* families. Combined expression of several of these expressed genes within lung cancers predicted an especially poor overall survival in lung cancer, as shown in Figure 6D. *KIF22* expression was associated with a statistically significant and unfavorable lung cancer survival when combined with increased *AURKA* expression levels, as displayed in Figure 6D.

**Figure 7:**
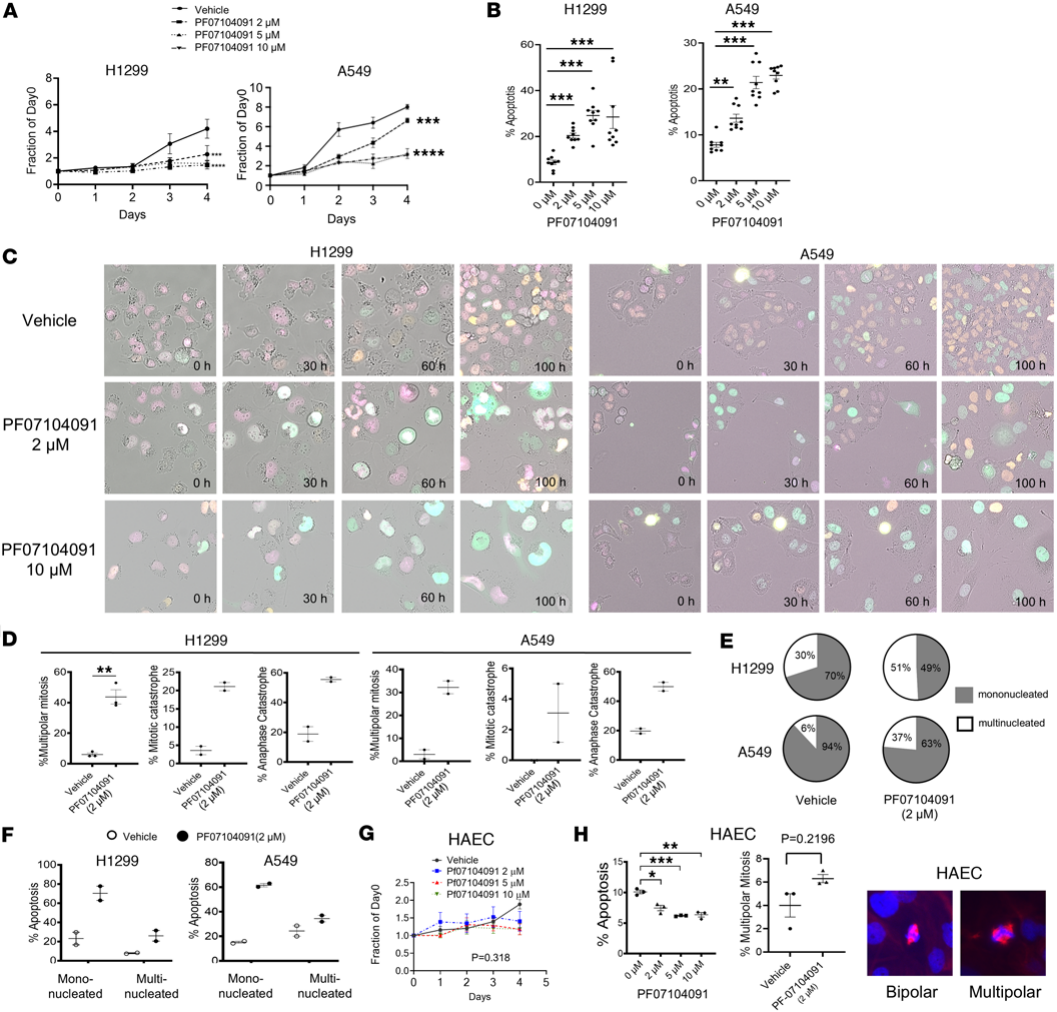
The effects of the CDK2 specific inhibitor PF07104091 on cancer cell proliferation, apoptosis, multipolar mitosis, and anaphase catastrophe. (**A**) PF07104091 treatment reduced proliferation in H1299 and A549 human lung cancer cells. (**B**) PF07104091 treatment increased the onset of apoptosis in both H1299 and A549 lung cancer cells. (**C**) Representative images of multipolar mitosis and G2 arrest in H1299 and A549 lung cancer cells were obtained after PF07104091 (2 μM or 10 µM) treatments as compared with vehicle controls. Each group was scored using time-lapse microscopic imaging. (**D**) PF07104091 treatment (5 μM for H1299 and 2 μM for A549) of lung cancer cells caused multipolar mitosis, mitotic catastrophe, and anaphase catastrophe. (**E**) PF07104091 treatment (5 μM for H1299 and 2 μM for A549) increased the ratios of multinucleated versus mononucleated lung cancer cells. (**F**) Multinucleated cancer cells had decreased apoptosis as compared with mononucleated lung cancer cells following multipolar mitosis events after PF07104091 treatment (5 μM for H1299 lung cancer cells and 2 μM for A549 lung cancer cells). (**G**) In marked contrast, PF07104091 treatment did not have a statistically significant effect on proliferation, apoptosis, or the onset of multipolar mitosis in primary human alveolar epithelial cells (HAECs). (**H**) PF07104091 (5 μM) treatment did not statistically significantly augment multipolar mitosis events in HAEC. Representative images of bipolar mitosis and multipolar mitosis are shown. Purple staining displayed γ-tubulin and blue signals indicated DAPI staining. The 1-way ANOVA test was used for **A** and **G** and 2-tailed Student’s *t* tests were used for **B** and **H**, with a Bonferroni correction of *P* value below 0.05 deemed statistically significant. Data are shown as mean ± SD, with the symbols indicating *P < 0.05, **P < 0.01, ***P < 0.001, and ****P < 0.0001, respectively. Total original magnification, ×40.

To further explore whether inhibition of CDK1 pathway or Kinesin family members repress proliferation of these multinucleated cancer cells that persist despite CDK2 inhibition, combined treatment of CYC065 with Ro-3306 (a CDK1 inhibitor) and independently with CYC065 with AZ82 (a KIFC1 inhibitor) were conducted in H1299 and ED1SQ4 cells. Combination treatment using subtherapeutic doses statistically significantly repressed growth of these lung cancer cells, as shown in Supplemental Figure 6. As expected, similar statistically significant results were observed with the CDK2 selective inhibitor PF07104091 when used in these combined regimens, as displayed in Supplemental Figure 6. The single agent activity of PF07104091 is presented in Figure 7.

### Specific CDK2 inhibition effects in aneuploid cancer cells.

To confirm that the observed effects were specific to CDK2 inhibition and not due to CDK2 off-target actions of CYC065 treatment, other CDK2 inhibitors that were even more selective for CDK2 than was CYC065 were examined. These agents were PF07104091 and AUZ545. PF07104091 is undergoing clinical trial testing for the treatment of solid tumors, including non–small cell lung cancers (NSCLC). PF07104091 and AUZ545 independently reduced proliferation and increased apoptosis response of H1299 and A549 lung cancer cells in a dose-dependent manner, as in Figure 7, A and B, and Supplemental Figure 7, A and B. Live-cell microscopy assays revealed that PF07104091 and AUZ545 each promoted multipolar mitotic events as compared with vehicle controls, as displayed in Figure 7, C and D, and Supplemental Figure 7C.

Mitotic events were rare at the high treatment concentration of 10 μM for PF07104091. This dosage predominately conferred cell cycle arrest at the G2/M phase, followed by cell death. PF07104091 and AUZ545 each triggered mitotic catastrophe and anaphase catastrophe as compared with vehicle treatment, as seen in Figure 7D and Supplemental Figure 7C. Notably, PF07104091 treatment also increased the proportion of multinucleated progeny following multipolar mitosis. The progeny subsequently entered a second multipolar mitosis, as in Figure 7E. These multinucleated cells were more resistant to apoptosis than were their mononucleated counterparts, as displayed in Figure 7F and Supplemental Figure 7C. To independently confirm the role of CDK2 inhibition in causing anaphase catastrophe, CDK2 targeting shRNA and control shRNA were independently transfected into H1299 lung cancer cells. Like the individual actions of CYC065 and PF07104091 treatments, CDK2 knockdown caused a statistically significant increase in multipolar mitosis, mitotic catastrophe, and anaphase catastrophe. Each of these events led to the onset of apoptosis, as shown in Supplemental Figure 7D. Immunoblot analysis confirmed the shRNA-mediated knockdown of CDK2, as shown in Supplemental Figure 7E.

Our prior work found that CDK2 inhibition did not appreciably trigger anaphase catastrophe in human immortalized bronchial epithelial cells or in primary HAEC (20, 21). Consistent with these findings, individual treatment with specific CDK2 inhibitors, such as PF07104091 or AUZ545, did not significantly reduce growth, induce apoptosis, or promote multipolar mitosis in HAECs when compared with vehicle-treated controls as established in Figure 7, G and H, and Supplemental Figure 7F. These results determine that CDK2 inhibition does not elicit multipolar mitosis or anaphase catastrophe in normal lung cells.

### Intravital imaging of lung cancer fates after CDK2 inhibitor treatment.

**Figure 8:**
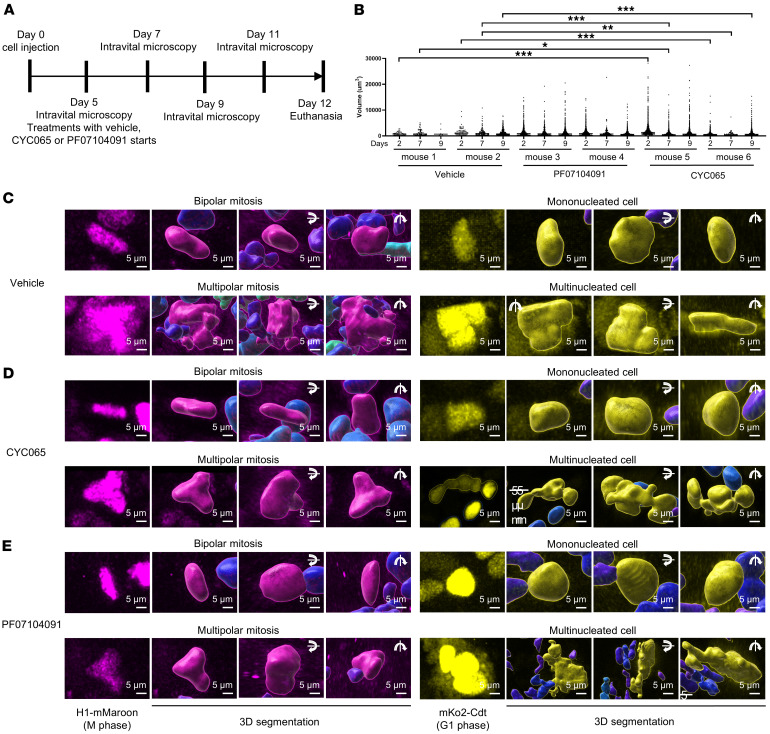
Intravital 2-photon microscopy of in vivo human H1299 lung cancers. (**A**) H1299 cells were transplanted into the tongues of individual NCG mice. Intravital microscopic images were acquired after individual CDK2 inhibitor-treatment versus controls for the indicated lengths of time. (**B**) Nuclear volumes were measured by segmentation of FUCCI mKO2 fluorescence signals. (**C**–**E**) Representative fluorescence images and 3D models of bipolar mitotic, multipolar mitotic, mononucleated, and multinucleated cellular events from each of the vehicle (**C**), CYC065 (**D**), and PF07104091 (**E**) treatment arms are shown. The Fisher’s exact test was used to compare nuclear volume distributions in intravital microscopy where a *P* value below 0.05 was deemed statistically significant. The symbols indicate *P < 0.05, **P < 0.01, and ***P < 0.001. Scale bars: 5 μm.

To confirm and extend these findings to the in vivo setting, intravital 2-photon microscopy was employed to assess whether CDK2 inhibition increased the presence of multipolar mitosis or promoted the onset of multinucleated cells. FUCCI probe–transduced H1299 cells were implanted into the tongues of NCG immunodeficient recipient mice, followed by independent CYC065, PF07104091, or vehicle treatment for 7 days, as summarized in Figure 8A. The implanted cancer cells were imaged based on the detected FUCCI fluorescence signals. CYC065- or PF07104091-treated tumors had a statistically significantly higher proportion of cells with large nuclear volumes versus vehicle treatments, as shown in Figure 8B. To independently confirm that the larger nuclear volumes arose from multinuclear cancer cells or cancer cells with multipolar mitosis, segmentation analysis was done to determine the precise characteristics of these cancer cells. Representative fluorescence images along with the corresponding 3D segmentation appear in Figure 8, C–E. Similar results were observed in syngeneic lung cancer cells that were implanted with FUCCI probe–transduced murine ED1SQ4 lung cancer cells before treatment with CYC065 or vehicle, as displayed in Supplemental Figure 8.

## Discussion

The objective of this study was to elucidate the biology of polyploid cells that arise after CDK2 inhibition in cultured normal HAEC and lung cancer cells as well as in PDX lung cancers. These findings reveal that CDK2 inhibition highlights a previously unrecognized conversion between bipolar mitosis, multipolar mitosis, and multinucleated cellular states. This confers a persistent cancer cell population, despite ongoing CDK2 treatment. These different cancer cell fates were detected in in vitro cultures and in in vivo PDX lung cancer models, as shown in Figure 1. However, these events were rarely detected in HAECs. This indicates that a therapeutic window exists between malignant versus normal lung tissues following CDK2 inhibition. These respective cancer cell fates were elucidated by use of transfected FUCCI cell cycle probes. This permitted the precise tracking of these diverse cellular fates following CDK2-inhibited populations and across different phases of the cell cycle. Hence, these findings provide a comprehensive view of the cellular fate outcomes after CDK2 antagonism that led to the loss of proliferation and the onset of apoptotic cell death, as detailed in Figure 2.

Another finding from this study is the emergence of multinucleated cancer cells that appeared after CDK2 inhibitor treatments. Intriguingly, these cancer cells continued to proliferate but with a reduced apoptotic rate that increased their survival as displayed in Figure 5. Using RNA-Seq analyses of 4N as compared with 2N populations, it was found that persistent cancer cells were enriched for CDK1-dependent pathways. This included in this population the overexpression of *AURKB*, *PLK1*, and many *KIF* family members. This enriched cluster identified in the polyploid cells was further analyzed using TCGA. The expression of these genes was associated with an unfavorable overall survival in patients with lung cancer, as shown in Figure 6. This indicated that the persistence of these multinucleated and multipolar cells revealed the presence of a residual population of aneuploid cancer cells despite continuous CDK2 inhibition. This population expressed genes that were associated with an unfavorable lung cancer survival. Hence, these findings have translational relevance. This observation indicates the need to find a way to eradicate this persistent population.

An intriguing aspect of this work was pursued using intravital 2-photon microscopy. This helped to extend studies from the in vitro to the in vivo setting of FUCCI-transduced lung cancer cells that were transplanted into recipient mice. These studies were performed using syngeneic murine lung cancer cells and independently with human lung cancer cells. The presented findings recapitulated the in vitro results in the in vivo setting. This demonstrated that CDK2 antagonism relative to controls caused a statistically significant increase in the population of cancer cells with larger nuclei indicative of their multinucleated or multipolar state, as seen in Figure 8. Since these multinuclear cancer cells proliferated and were resistant to apoptosis caused by CDK2 inhibition, these experiments indicate the potential for CDK2-inhibited cells to persist and grow because of their capacity to transition between different cellular states, each with distinct biological properties. This work highlights that multiple cell death programs exist following CDK2 inhibition. These include the onset of mitotic catastrophe when the parental cancer cells divide as well as the appearance of anaphase catastrophe when the progeny enter mitotic division.

The findings presented here indicate that, despite CDK2 antagonism of cultured or in vivo lung cancer PDXs, there exists a residual population of aneuploid cancer cells that continue to proliferate and resist apoptosis. These observations are translationally relevant since this persistent population likely contributes to the inability of CDK2 inhibition as a single agent to eliminate lung tumor growth. This could account for the relatively modest antineoplastic effects that have been observed to date in the clinic with CDK2 cell cycle inhibitors when used as single agents (15).

One way to eliminate this persistent cancer cell population is to explore combination therapy with CDK2 inhibitors in concert with another centrosome targeting agent. Since we found that CDK2 antagonism opposed centrosome clustering (18–21), combining such an inhibitor with a PLK4 inhibitor that we reported to promote supernumerary centrosomes (46) could reduce proliferation and augment apoptosis in polyploid cancer cells.

In support of this concept was our prior work that revealed synergistic antineoplastic effects with a first-generation CDK2 inhibitor combined with the PLK4 inhibitor CFI-400945 (46). Another promising strategy to consider involves use of TTK (MPS1) inhibition, which affects the spindle assembly checkpoint (SAC) and leads to excessive chromosomal instability and mitotic failure (47). The combined treatment of a TTK inhibitor with a CDK2 antagonist might eradicate persistent cancer cells that follow single-agent CDK2 inhibitor treatment as reported in this study. Dual inhibition of CDK2 and CDK1 activity was explored to prevent polyploid cancer cells from reentering the cell cycle, forcing them into eliciting an apoptotic death program (39). CDK4/6 inhibitors have been proposed for use in combination therapy with a CDK2 inhibitor to prevent compensatory upregulation of CDK4/6 activity, allowing cancer cells to bypass the G1/S checkpoint (48). Targeting Aurora Kinase A along with CDK2 inhibition should destabilize spindle dynamics, increasing mitotic errors and eliminating polyploid tumor cells (49). Other approaches could include combining a CDK2 antagonist with an ATR or CHK1 inhibitor to disrupt DNA damage response pathways. This would lead to replication stress–induced lethality, and BCL2/MCL1 inhibitors would augment apoptotic priming (50). Further support for the therapeutic potential in lung cancer of using a CDK2 inhibitor combined with either a CDK1 or KIFC1 inhibitor is provided in Supplemental Figure 6.

Taken together, the preclinical findings reported here provide a rationale for the design and execution of future clinical trials in the lung cancer clinic that would dually target centrosome stoichiometry. We hypothesize that this regimen will cooperatively eliminate persistent polyploid cancer cells by promoting apoptotic death.

## Methods

### Sex as a biological variable.

This study examined antineoplastic effects of CDK2 inhibition in PDX tumors derived from both male and female patients with lung cancer. Similar CDK2 inhibitor antineoplastic effects were observed in lung cancer PDXs derived from both sexes. This study used female NSG mice as recipients for lung PDX tumors, female FVB/N mice for the ED1SQ4 syngeneic model, and female NCG mice for the H1299 xenograft model because female recipient mice typically exhibit less aggressive behaviors than do male mice. Studies performed in syngeneic and xenograft lung tumors did not reveal an appreciable difference in antitumor responses to CDK2 inhibitors observed between male and female recipient mice (data not shown).

### Cell culture.

CYC065 was obtained from the Division of Cancer Treatment and Diagnosis (DCTD), National Cancer Institute (NCI). Tagtociclib (PF07104091) (catalog S9878), AUZ454 (catalog S8100), and Ro-3306 (catalog S7747) were each purchased from Selleckchem. AZ82 (catalog HY-12241) was purchased from MedChemExpress. Human lung cancer cell lines H1299, A549, HOP62, and H522 cancer cell lines were purchased and authenticated by American Type Culture Collection (ATCC). Murine lung cancer cell lines ED1SQ4 and 344SQ cells were independently derived from lung cancers arising from cyclin E–overexpressing transgenic mice or Kras^LA1/+^ engineered mice, respectively, and were authenticated, as previously described (46, 51–53).

To generate FUCCI-expressing cell lines, viral particles were produced by transfection of the psPAX2 (12260, Addgene), pMD2.G (12259, Addgene), Clover-Geminin(1-110)-IRES-mKO2-Cdt(30-120) (83841, Addgene), and mTurquoise2-SLBP(18-126)-IRES-H1-mMaroon1 (83842, Addgene) with Lipofectamine 3000 (L3000001, GeneCopoeia) into HEK293T cells. Target cells were transduced with the desired viral supernatant containing 10 g/mL Polybrene (TR-1003-G, MilliporeSigma) before sorting by flow cytometry for purification. These cells were cultured in RPMI 1640 media supplemented with 10% FBS, antimycotics, and antibiotics at 37°C with 5% CO_2_ within a humidified incubator. Mycoplasma was screened for using the MycoAlert Mycoplasma Detection Kit (LT07-318, Lonza). All experiments were conducted using cells that tested mycoplasma negative.

### Primary HAECs.

Primary HAECs were purchased and authenticated by the vendor (H-6053, Cellbiologics). These cells were cultured in Complete Human Epithelial Cell Medium (H6621, Cellbiologics) supplemented with Insulin-Transferrin-Selenium (41400045, Thermo Fisher Scientific), epidermal growth factor (AF-100-15-500UG, Thermo Fisher Scientific), hydrocortisone (S003K, Thermo Fisher Scientific), and antibiotic-antimycotic solution (15240062, Thermo Fisher Scientific) in 10% FBS at 37°C with 5% CO_2_ within a humidified incubator. Mycoplasma testing was with the MycoAlert Mycoplasma Detection Kit (LT07-318, Lonza).

### Plasmids, shRNAs, and transfection.

Plasmids shCDK2 (HSH151382-LVRH1MH, Genecopoeia) and empty vector (CSHCTR001-LVRH1MP, Genecopoeia) were purchased. Lentiviral particles were produced by transfection of HEK293T cells with the shCDK2 or empty vector plasmid, psPAX2 (12260, Addgene) and pMD2.G (12259, Addgene) with Lipofectamine 3000 (L3000001, GeneCopoeia). Exponentially growing H1299 lung cancer cells were transduced with the viral supernatant containing 10 µg/mL Polybrene (TR-1003-G, MilliporeSigma) and then sorted for purification by flow cytometry. Transduction efficiency was determined by measuring mCherry fluorescence signals using fluorescence microscopy and by protein expression using immunoblot assays.

### Lung cancer PDX models.

Lung cancer PDXs were established from lung cancer clinical specimens that were surgically resected at the University of Texas MD Anderson Cancer Center (Houston, Texas, USA) (54, 55). Tumors were cut and implanted into the flank s.c. space of athymic nude or NSG mice (005557, The Jackson Laboratory). These mice were treated with vehicle or with CYC065 (50 mg/Kg) for 21 days. Tumors were resected, and the final tumor weight was measured. The current study extended our prior work (20) by examining the histopathology of lung PDXs at the point that maximum antitumor effects were observed after treatment of tumor-bearing mice with CYC065 as compared with controls. Representative changes in the growth of lung PDXs are shown.

### IHC.

Lung cancer xenografts were fixed with 10% formalin immediately after resection and were then paraffin embedded. IHC staining and image acquisition were performed by Vitro-Vivo Biotech. Bipolar or multipolar mitotic cells were determined by phospho-histone H3 (Ser10) antibody (9701, Cell Signaling Technology) and hematoxylin staining. Polyploid and multinucleated cells were visualized based on H&E staining.

### Immunoblot assays.

Cells were lysed with ice-cold Pierce RIPA Lysis and Extraction Buffer (89900, Thermo Fisher Scientific) supplemented with Halt Protease and Phosphatase Inhibitor Cocktail (78440, Thermo Fisher Scientific). Proteins were resolved by SDS-PAGE before transfer to Trans-Blot Turbo Mini 0.2 μm PVDF Transfer Packs (1704156, Bio-Rad). Membranes were blocked with 5% nonfat milk in Tris-buffered saline (1706404XTU, Bio-Rad) and with 0.1% Tween-20 (170653, Bio-Rad) (TBS-T) solution for at least 1 hour before overnight incubation at 4°C with a primary antibody diluted in 1% nonfat milk or 1% BSA in TBS-T. This was followed by 10-minute washes 3 times in TBS-T solution and with a 1-hour incubation with the desired secondary antibody diluted in 5% nonfat milk. After 3 additional washes, antibody binding was visualized by Clarity Western ECL Substrate (1705061, Bio-Rad) and quantified by ImageLab software (Bio-Rad). Primary antibodies independently used were: CDK2 (ab32147, Abcam), GAPDH (14C10, Cell Signaling Technology), and β-actin (4967, Cell Signaling Technology). Secondary goat anti–mouse IgG (1706515, Bio-Rad) and goat anti–rabbit IgG (1706516, Bio-Rad) were used. Immunoblots were stripped using Restore PLUS Western Blot Stripping Buffer (46430, Thermo Fisher Scientific) followed by TBS-T solution washing.

### Cell viability assay.

Exponentially growing cells were plated onto 6-well culture dishes plated at 50 × 10^3^ to 100 × 10^3^ cells per well and treated with vehicle (dimethyl sulfoxide, DMSO) or CYC065 (0.2 µM). Cells were supplemented with fresh media containing CYC065 (0.2 µM) on day 0 and continued throughout the 4 days of the experiment. At the indicated time points cell viability was measured by Trypan blue (TB) dye staining. Viable cells (TB negative) were counted, and the numbers of dead cells (TB positive) were excluded from the final score. Experiments were done at least in triplicate independent replicates.

Exponentially growing ED1SQ4 or H1299 lung cancer cells stably expressing GFP were seeded at optimized densities onto 96-well tissue culture plates in triplicate. Cells were independently treated with vehicle, CYC065, Ro-3306, or AZ82 or with combinations at varying dosages 24 hours later. Proliferation was measured after an additional 24–96 hours of treatments with the indicated drug exposures using live cell counting by the Celigo Instrument (Celigo). Experiments were done at least in replicate experiments, each done in triplicates.

### Proliferation assays.

Exponentially growing cells were seeded at optimized densities for each examined cell line plated onto individual wells of 12-well tissue culture dishes, in triplicate. Cells were treated with CYC065 at varying dosages and independently with the vehicle (DMSO) as a control, 24 hours later. Proliferation was measured using the WST-1 assay (5015944001, MilliporeSigma) after 48–72 hours of the indicated drug exposures. Proliferation studies were independently replicated at least 3 times. For proliferation assays that assessed combinations with CDK2 inhibitors and CDK1 or KIFC1 inhibitors, experiments were done in triplicate and were replicated at least twice to confirm results.

### Live cell imaging assays.

Cells were plated onto glass-bottom plates in RPMI 1640 media supplemented with 10% FBS with antimycotics and antibiotics added. Cells were allowed to adhere for 24 hours. Cells were then cultured in the sterile microscope chamber maintained at 37°C and 5% CO_2_. Fluorescence images were acquired every 15 minutes by a time lapse imaging system for 96 hours using a Axiocam 506 color microscope and the Live-Cell Imaging system (Carl Zeiss Microscopy). Cells exhibiting bipolar mitosis, multipolar mitosis, multinuclear divisions, failed cytokinesis, or cell fusion events were each scored.

### Apoptosis analysis by live cell imaging assays.

Exponentially growing H1299 and A549 lung cancer cells were plated in vehicle or CYC065 (0.2 µM) containing RPMI 1640 media supplemented with 10% FBS and with antimycotics and antibiotics. Staurosporin (100 nM; 9953, Cell Signaling Technology) treatment was used as a positive control for apoptosis induction. Live-cell apoptosis detection dye CF 647 Annexin V Conjugate (29003R, Biotium) was supplemented in the media. Cells were cultured in the Axiocam 506 color microscope chamber (Carl Zeiss Microscopy) at 37°C and with 5% CO_2_. Apoptotic (annexin V^+^) cells were scored based on the presence in the studied cells of blue subnuclear staining.

### Volume electron microscopy.

Correlative Light and Electron Microscopy (CLEM)/FIB-SEM studies were done using previously described methods (21, 56). Indicated cancer cell lines were seeded on alphanumerically coded gridded glass cover slipped wells (P35G-1.5-14-C-GRD, MatTek) and processed for FIB-SEM using a modified protocol (57). Image acquisition was conducted using Zeiss Crossbeam 550 FIB-SEM (21). A typical run generated approximately 2,000 two-dimensional (2D) images. FIB-SEM reconstructions were analyzed using IMOD (version 4.7) and 3DSlicer (version 4.6) software. The slicer module in IMOD captured 2D images in acquisition and arbitrary planes. Segmentation assignments were aided by checking the accuracy of structures in all 3 planes (*x*, *y*, and *z*) for FIB-SEM image planes. The 3DSlicer was used for visualization and generation of merged FIB-SEM/3D segmentation images.

### RNA-Seq analysis.

ED1SQ4 lung cancer cells treated with vehicle as a control or CYC065 (0.5 µM) for 72 hours were stained with Vybrant DyeCycle Green Stain (V35005, Thermo Fisher Scientific) for 1 hour. Cells were then washed with phosphate buffered saline (PBS) and then sorted for diploid and for polyploid populations by S3e Cell Sorter (Bio-Rad) based on DNA quantification.

Total RNA was purified using RNeasy Plus Universal Kit (73404, Qiagen). The Illumina Stranded Total RNA library preparation ligation with RiboZero plus (20040526, Illumina) was used for RNA-Seq. Ribosomal RNA (rRNA) was removed using biotinylated target-specific oligos with the Ribo-Zero Plus rRNA Depletion Kit (20037135, Illumina). RNA was fragmented and used to generate first strand cDNA using reverse transcriptase and random primers, followed by second-strand cDNA synthesis using DNA Polymerase I and RNase H. The resulting double-stranded cDNA was used as the input to a standard Illumina library preparation with end-repair, adapter ligation, and PCR amplification to yield a library for sequencing. The final purified product was quantitated by qPCR assays before cluster generation and sequencing on NextSeq 2000 P2 flowcell (Illumina) for paired-end 100-cycle sequencing.

The Illumina bcl2fastq (version 2.20) was used to demultiplex and convert binary base calls and qualities to fastq format. The sequencing reads were trimmed to remove adapters and low-quality bases using Cutadapt (version 1.18). The trimmed reads were mapped to mouse reference genome (mm10) and GENCODE annotation M21. The mapping was done using STAR aligner (version 2.7.0f) with a 2-pass alignment option. RSEM (version 1.3.3) was used for gene and transcript quantification based on the GENCODE M21 GTF file. To identify differentially expressed genes (DEGs), DESeq2 (version 1.44.0) was used. Volcano plots were generated using EnhancedVolcano (https://github.com/kevinblighe/EnhancedVolcano; commitID: ab0428ef29e2a3bf35aec63d4eeb8f9302963645). Heatmap plots were generated using pheatmap (https://github.com/raivokolde/pheatmap; commitID: b33345349662ad3cbfae696b2df91aecfa8cefd5). DEGs were scored using these criteria: adjusted P < 0.05 and fold change log_2_FC > 1. The statistically significant DEGs were further analyzed using Metacore software (version 24.1, https://clarivate.com/products/metacore/) to perform pathway enrichment analyses and to generate pathway maps.

### qPCR assays.

qPCR assays were performed as previously described (21). The respective primers used were: murine cyclin B1 primer (Mm03053893_gH); murine Cdc25c primer (Mm00486872_m1); murine Cdc20 primer (Mm07297857_g1); murine Kif18a primer (Mm01327658_m1); murine Kif2c primer (Mm00728630_s1); murine Kifc1 primer (Mm00835842_g1); murine Aurka primer (Mm01248177_m1); murine cyclin A2 primer (Mm00438063_m1); murine Cdk1 primer (Mm00772472_m1); murine Plk1 primer (Mm00440924_g1); and murine β-actin primer (Mm02619580_g1).

### Bioinformatic analysis.

Bioinformatic analysis using TCGA database was conducted using corporals web-based tools (https://www.cbioportal.org/). RNA-Seq data of lung adenocarcinomas from PanCancer Atlas (566 patients) were interrogated. The mRNA expression *z* scores relative to normal samples (log RNA Seq V2 RSEM) were analyzed. All these expressed genes (CCNB1, CCNA2, CDK1, PLK1, AURKA, CDC25c, CDC20, KIF18A, KIF2C, KIFC1) were defined by cBioPortal as “altered” and “unaltered” versus normal expression levels. Distributions of overall survival were compared using the Kaplan-Meier method and log-rank tests.

### Immunofluorescence assays.

Human H1299 and murine ED1SQ4 lung cancer cells were fixed in ice-cold methanol (176845000, Thermo Fisher Scientific) and stained with β-actin antibody (66009-1-Ig, Proteintech), caspase 3/7 antibody (C10423, Thermo Fisher Scientific), Lamin B1 antibody (ab133741, Abcam), and Hoechst 33342 (62249, Thermo Fisher Scientific). Cells were scored for caspase^+^ staining and for mononucleated or multinucleated cells.

Primary HAEC were fixed in ice-cold methanol and stained with α-tubulin antibody (T6199, Sigma-Aldrich) along with Hoechst staining and mounted with Pro-Long Gold antifade reagent (P36934, Invitrogen). Secondary antibodies were Texas red anti-murine IgG (H+L) (TI-2000, Vector Laboratories) and goat anti–rabbit IgG (H+L) Cross-Adsorbed Alexa Fluor 488 (A11008, Thermo Fisher Scientific), respectively. Bipolar and multipolar mitotic events were scored using a Axiocam 506 color microscope with the LSM 900 Confocal system (Carl Zeiss Microscopy LLC). Each assay was performed in triplicate. Independent biologic replicate experiments were done at least in triplicates.

### Intravital microscopy of lung cancer models.

FUCCI transduced ED1SQ4 (50 × 10^3^ cells/injection) and H1299 (500 × 10^3^ cells/injection) were independently injected into the tongues of FVB/N and NCG mice, as previously described (58). Animals were treated with vehicle (60% Phosal [NC0130871, Thermo Fisher Scientific], 40% PEG400 [202398, MilliporeSigma], and 10% EtOH [BP2818100, Thermo Fisher Scientific]), 50 mg/kg of CYC065 or 200 mg/kg PF07104091, administered daily by oral gavage. On days 3 and 5 of treatments, mice were anesthetized by brief exposure to isoflurane followed by s.c. injection of a mixture of 100 mg/kg ketamine and 10 mg/kg xylazine (AnaSed LA, VetONE). Tongues were gently extended and stabilized with cotton tips on the 37°C heated stage of a 2-photon microscope. Hydration was carefully maintained by applying a thin layer of carbomer 940 gel with gauze wetted with distilled water placed over the eyes.

Mice were covered with a blanket during the experiment to maintain their comfort and physiological temperatures. Imaging was with an inverted TCS SP8 Dive Spectral Microscope (Leica) equipped with Mai-Tai and Insight X3 tunable lasers (Spectral Physics) and a 37°C preheated 40× objective (NA 1.10, HC PL IRAPO, Leica). Tissues were excited at 920 nm and 1,025 nm simultaneously. The mKO2 signal was detected by HyD-RLD3 at the emission of 560–590 nm; mMaroon1 was detected by HyD-RLD4 at the emission of 640–701 nm. The tile image of each tumor was collected by bidirectional line scan with 2 lines averaging at 600 Hz (320 × 320 pixel; 12 bits per pixel) on XY and 75–100 Z steps (2 mm step size) and stitched using LAS X Navigator. To image a large tumor in 3D volume, images were acquired in grids/tiles on XY to a maximum Z depth of about 200 µm. The settings used were bidirectional line scan with 2 lines averaging at the speed of 600 Hz (320 × 320 pixel; 12 bits per pixel) and 75–100 Z steps with 2 mm per step size. The grids were stitched together to render a large 3D tumor using LAS X Navigator software for quantification. The 3D images were acquired by bidirectional line scanning with 2 lines averaging at 400 Hz (512 × 512 pixel; 12 bits per pixel) on XY and 60–66 Z-stacks (2 μM step size) using Leica LAS X software. All images were stored as Leica Image Files (LIF) and processed further using Imaris (Bitplane). Nuclear volumes were measured by a segmentation algorithm in Imaris.

### Statistics.

Statistical analyses were done with SPSS Statistics software (version 23, SPSS) and with GraphPad Prism software (version 8, GraphPad Software). Data were displayed as mean ± SD. Two-tailed Student’s *t* tests compared differences between study groups with a *P* value below 0.05 deemed statistically significant. One-way ANOVA compared the differences of proliferation between vehicle and PF07104909 treated cancer cells. Fisher’s exact test was used to compare nuclei volume distributions in intravital microscopy where a *P* value below 0.05 was deemed statistically significant. Bonferroni corrections were used for multiple comparisons. Results of independent experiments were pooled to assess statistical significance. Experiments were typically performed in triplicate with at least 3 independent biological replicate experiments. Differential gene expression analysis was performed using a negative binomial model and Wald test with Benjamini-Hochberg correction.

### Study approval.

All experiments using animals were performed in accordance with *Guide for the Care and Use of Laboratory Animals* (National Academies Press, 2011) and were compliant with all relevant ethical regulations regarding animal research. Correlative scientific studies were previously approved by the MD Anderson Cancer Center IRB. All experiments were conducted following an IACUC-approved protocol.

### Data availability.

The datasets used as well as analyzed for this study will be available from the corresponding author upon reasonable request. The RNA-Seq data are available at the National Center for Biotechnology Information (NCBI) Gene Expression Omnibus (GEO) database (GSE292967; https://www.ncbi.nlm.nih.gov/geo/query/acc.cgi?acc=GSE292967). Values for all data points in graphs are reported in the Supporting Data Values file.

## Author contributions

ED and Xi Liu conceived the study design, supervised the study, and interpreted the data. LTM and BL interpreted the data. LTM, ZC, Xiuxia Liu, MK, AB, SCO, BO, YA, YN, RW, AH, and KN, collected data. Xi Liu, LTM, and ED wrote this manuscript.

## Supplementary Material

Supplemental data

Unedited blot and gel images

Supporting data values

## Figures and Tables

**Table 1:**
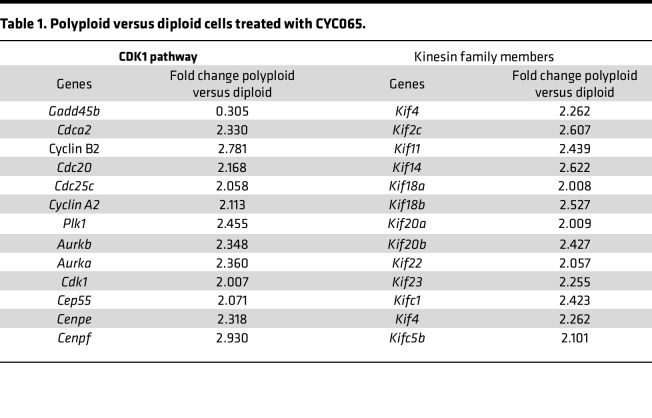
Polyploid versus diploid cells treated with CYC065.

**Table 2:**
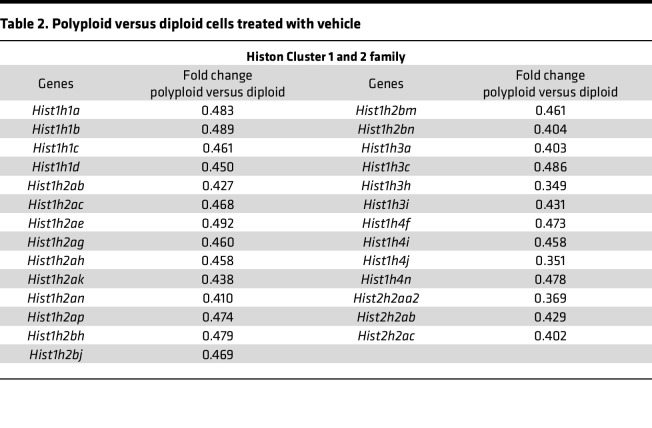
Polyploid versus diploid cells treated with vehicle

## Version 1 04/15/2025

In-Press Preview

## Version 2 05/22/2025

Electronic publication
