# Supplementary material for: CDK2 inhibition produces a persistent population of polyploid cancer cells
Source: JCI Insight. 2025 Apr 15;10(10):e189901. doi: 10.1172/jci.insight.189901 (PMC12128980; doi:10.1172/jci.insight.189901)
Supplement: Supplemental data [file jciinsight-10-189901-s205.pdf]

Supplementary Fig 1

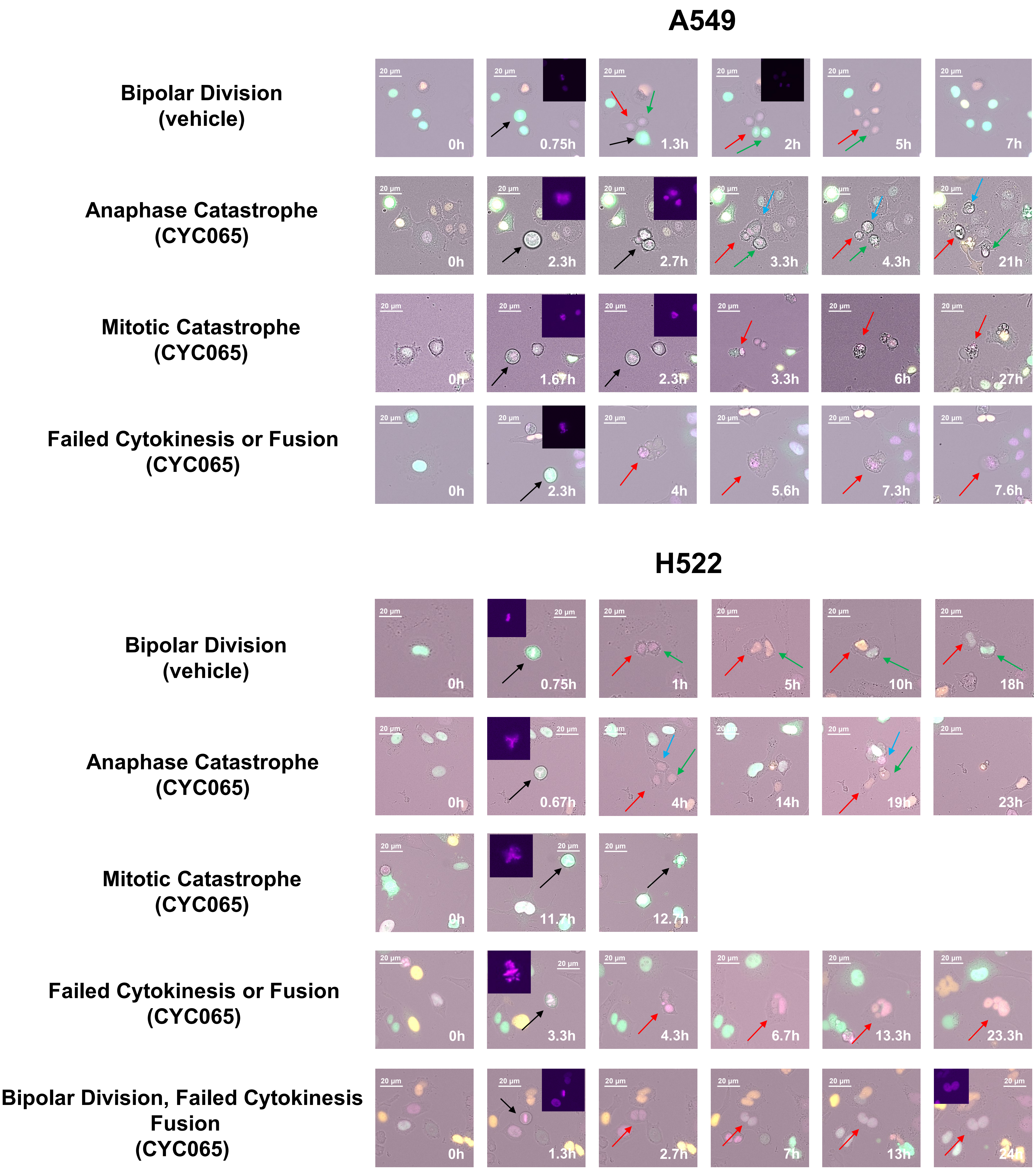

Supplementary Figure 1: Fates of lung cancer cells after CDK2 antagonism with CYC065-treatment. The fates of the indicated lung cancer cells were monitored by live cell microscopy using Fluorescent Cell Cycle Indicator (FUCCI) probes. Mitotic events of A549 and H522 lung cancer cells that stably expressed FUCCI vectors were treated with CYC065 or vehicle to interrogate bipolar and multipolar divisions. Indicated cells were monitored for 96 hours by time lapse microscopy. Representative images of the fates of these cells were shown for multipolar anaphases and included failed cytokinesis, mitotic catastrophe, and anaphase catastrophe, respectively.

Supplementary Fig 2

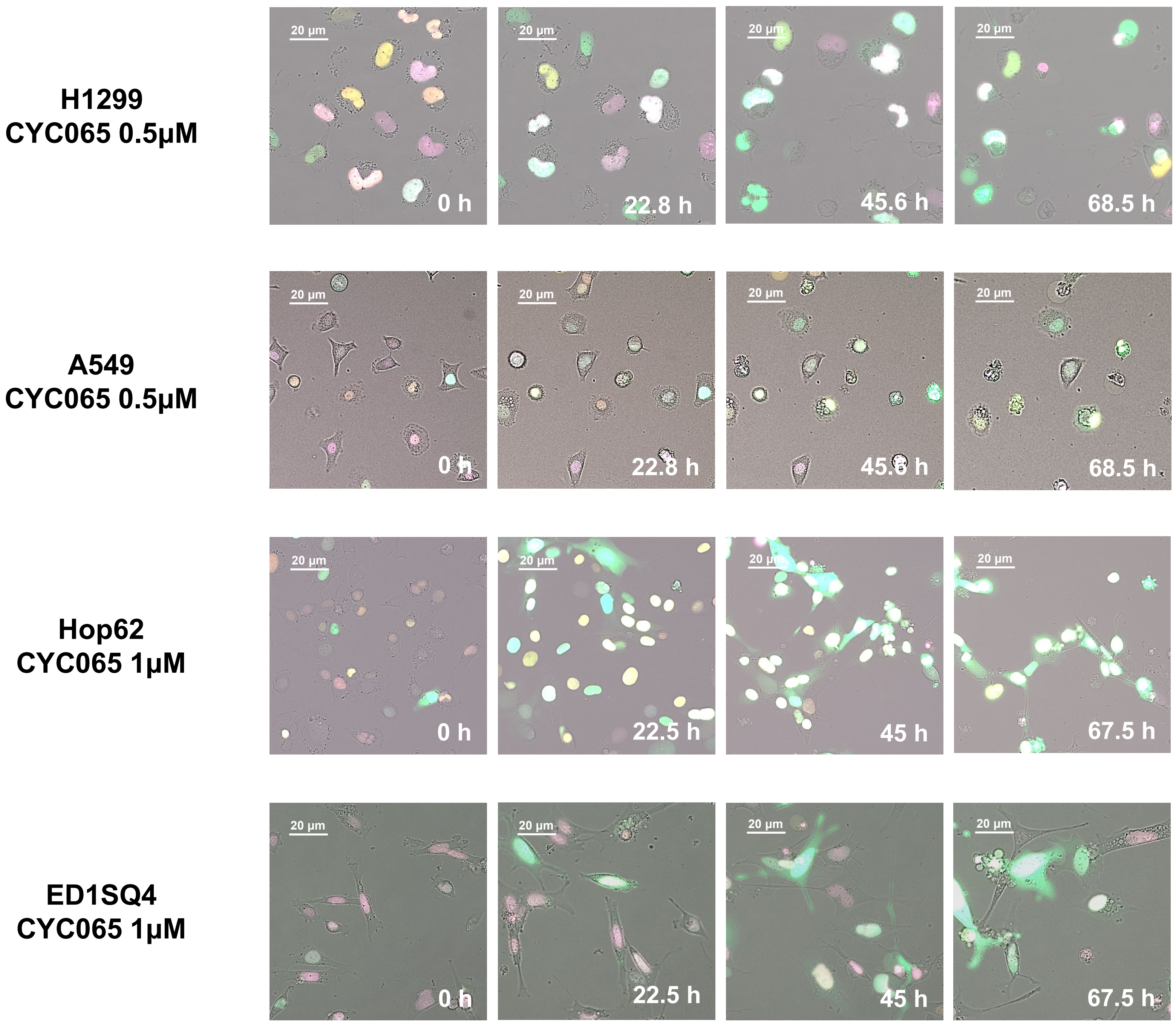

Supplementary Figure 2: Effects of high concentrations of CYC065-treatment caused G2/M cell cycle arrest followed by cell death. Representative images show H1299, A549, Hop62 and ED1SQ4 lung cancer cells individually observed over a period of 96 hours using time lapse microscopy. Apoptotic cells appear at the final time points of these time courses. Yellow fluorescence (mKO) and green fluorescence (Geminin) measured the onset of G0/G1and G2/M cell cycle phases, respectively. The purple stain (Annexin V) indicated the occurrence of apoptosis.

Supplementary Fig 3

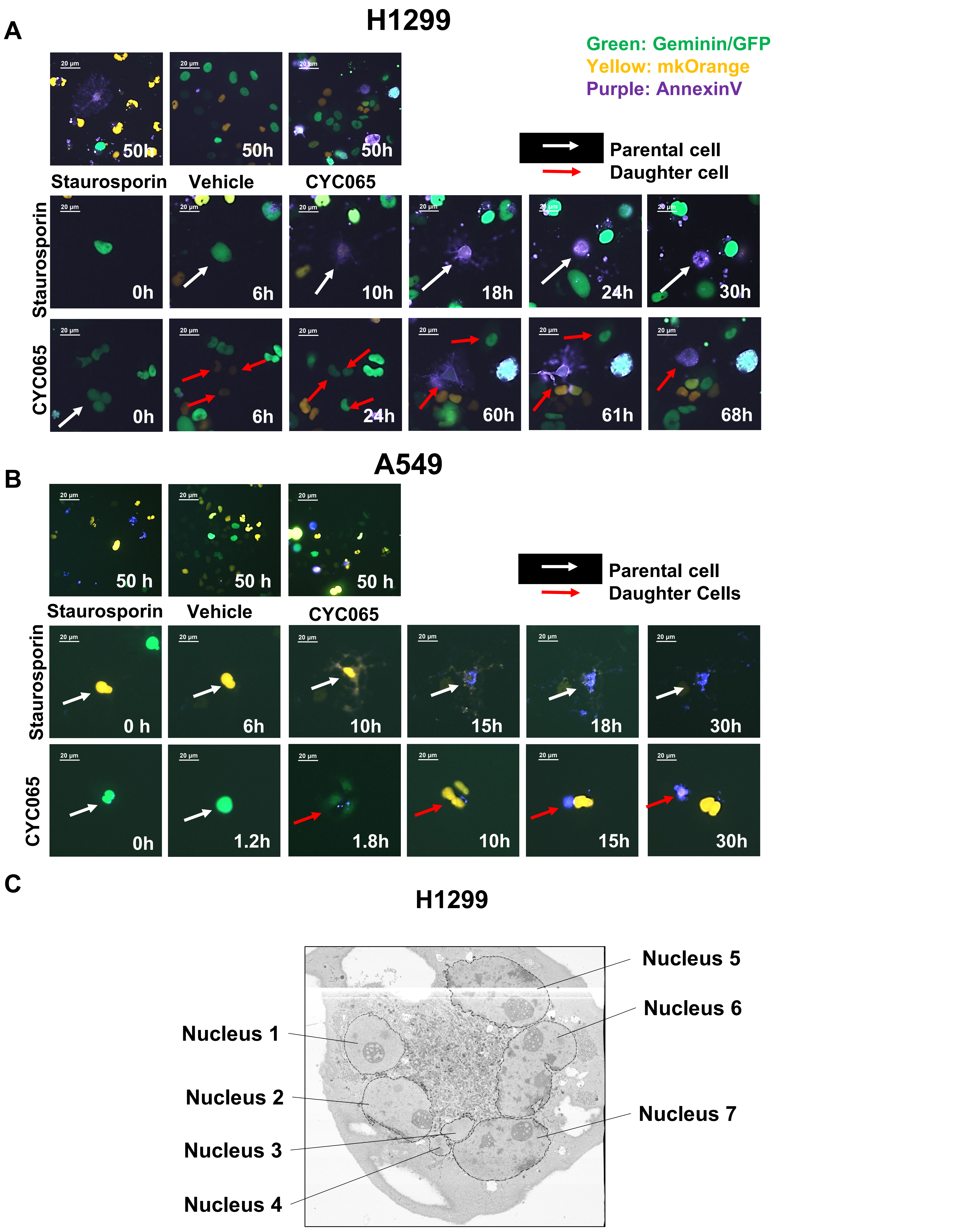

Supplementary Figure 3: Anaphase catastrophe is a pro-apoptotic mechanism. Live cell dye staining indicated Annexin V positivity in the progeny cells monitored for 96 hours after mitotic events. This indicates that cell death in multipolar mitotic progeny led to an apoptotic response. The (**A**) H1299 and (**B**) A549 human lung cancers cells were individually treated with Staurosporin (100nM) as a positive apoptotic control, vehicle or CYC065 and monitored by live cell time lapse microscopy for 96 hours. Yellow fluorescence (mKO) corresponds to the G0/G1 stage of the cell cycle and Green fluorescence (GFP) corresponds to the G2/M phase. Blue staining (AnnexinV) appears in dying cells and coincides with changes in cell structure and appearance that are morphologically consistent with the onset of apoptosis. (**C**) A representative FIB-SEM image is shown of a multinucleated H1299 lung cancer cell line that was treated with CYC065 (0.2µM).

Supplementary Fig 4

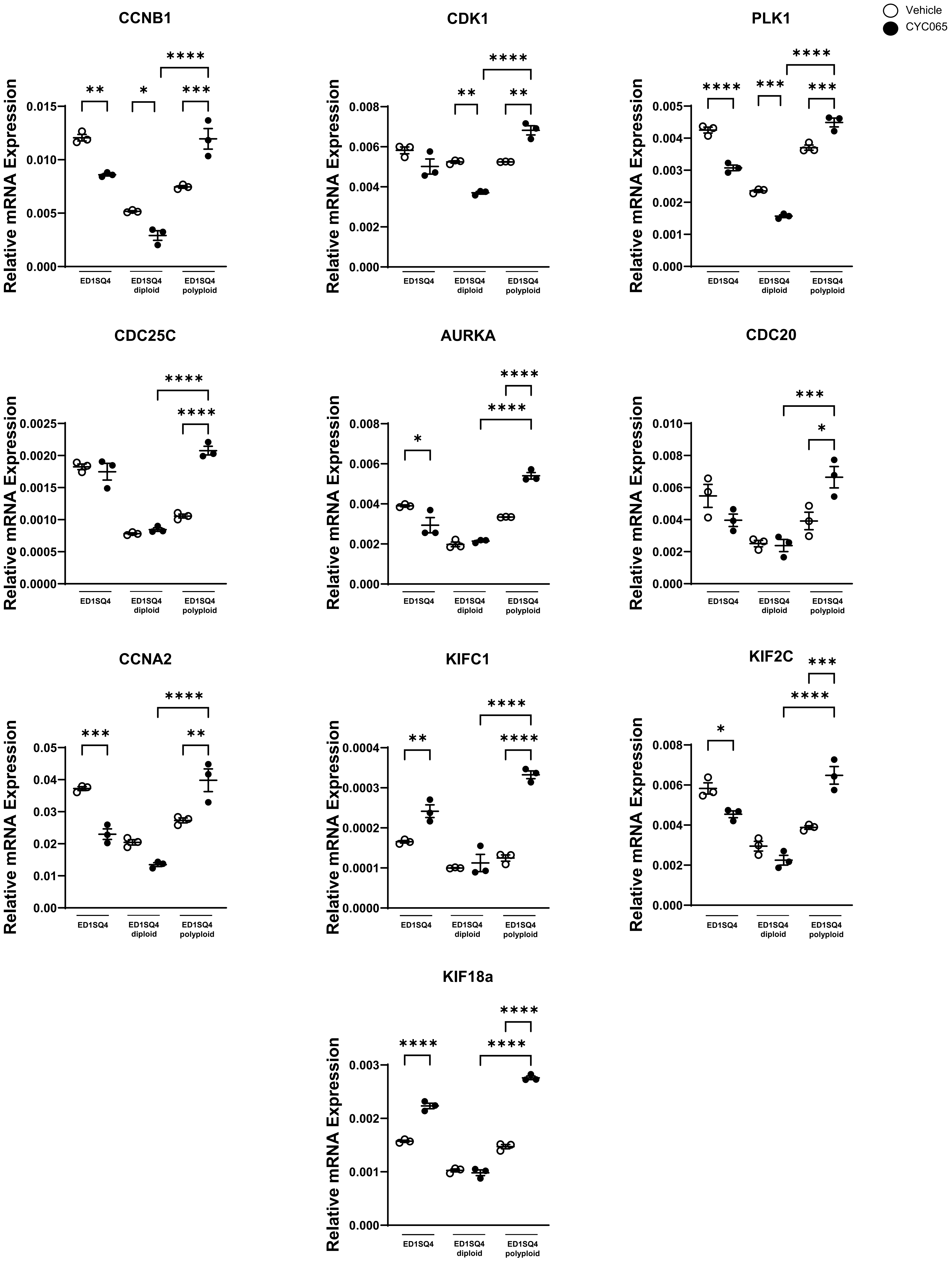

Supplementary Figure 4: Real-time PCR assays validated the representative differentially expressed species (Cyclin B1, Cdk1, Plk1, Cdc25c, Aurora Kinase A, Cdc20, Cyclin A2, KIFC1, KIF2C, and KIF18a) in parental versus polyploid versus diploid ED1SQ4 lung cancer cells after CYC065 treatments (as compared to vehicle controls). Error bars represented standard deviations with the symbols indicating * $P < 0.05$, ** $P < 0.01$, *** $P < 0.001$ and **** $P < 0.0001$, respectively.

Supplementary Fig 5

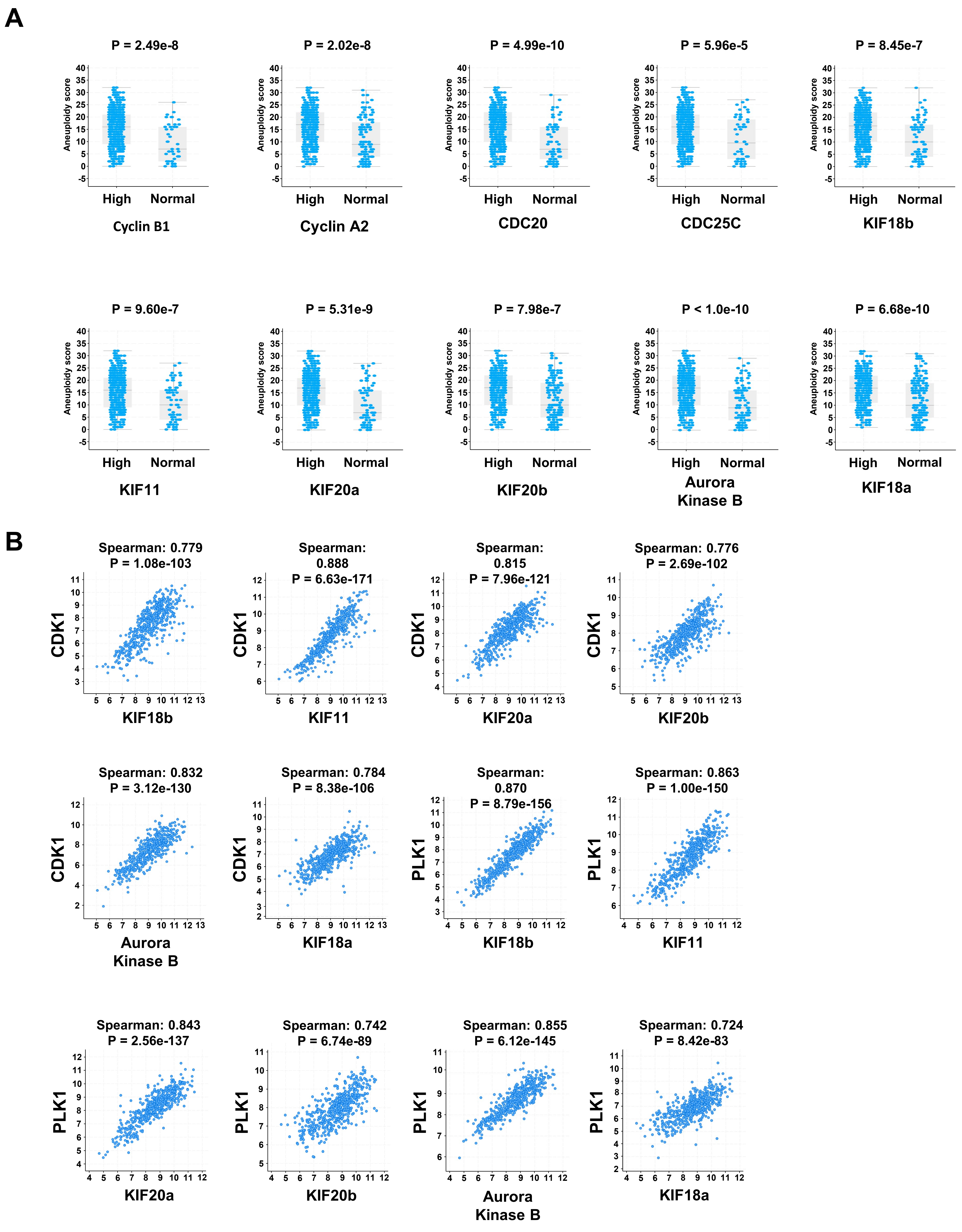

Supplementary Fig 5 Continued

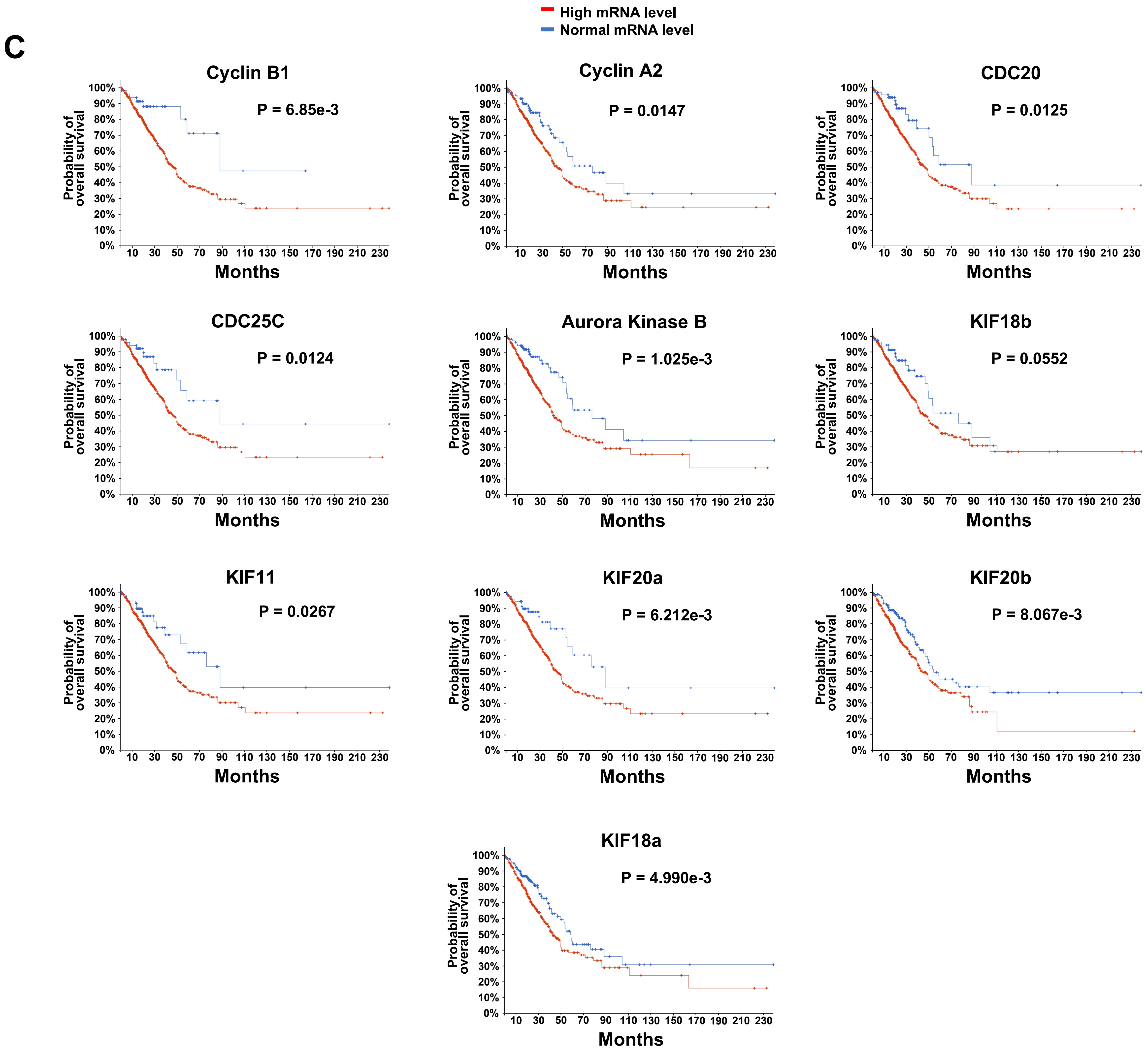

Supplementary Fig 5 Continued

D

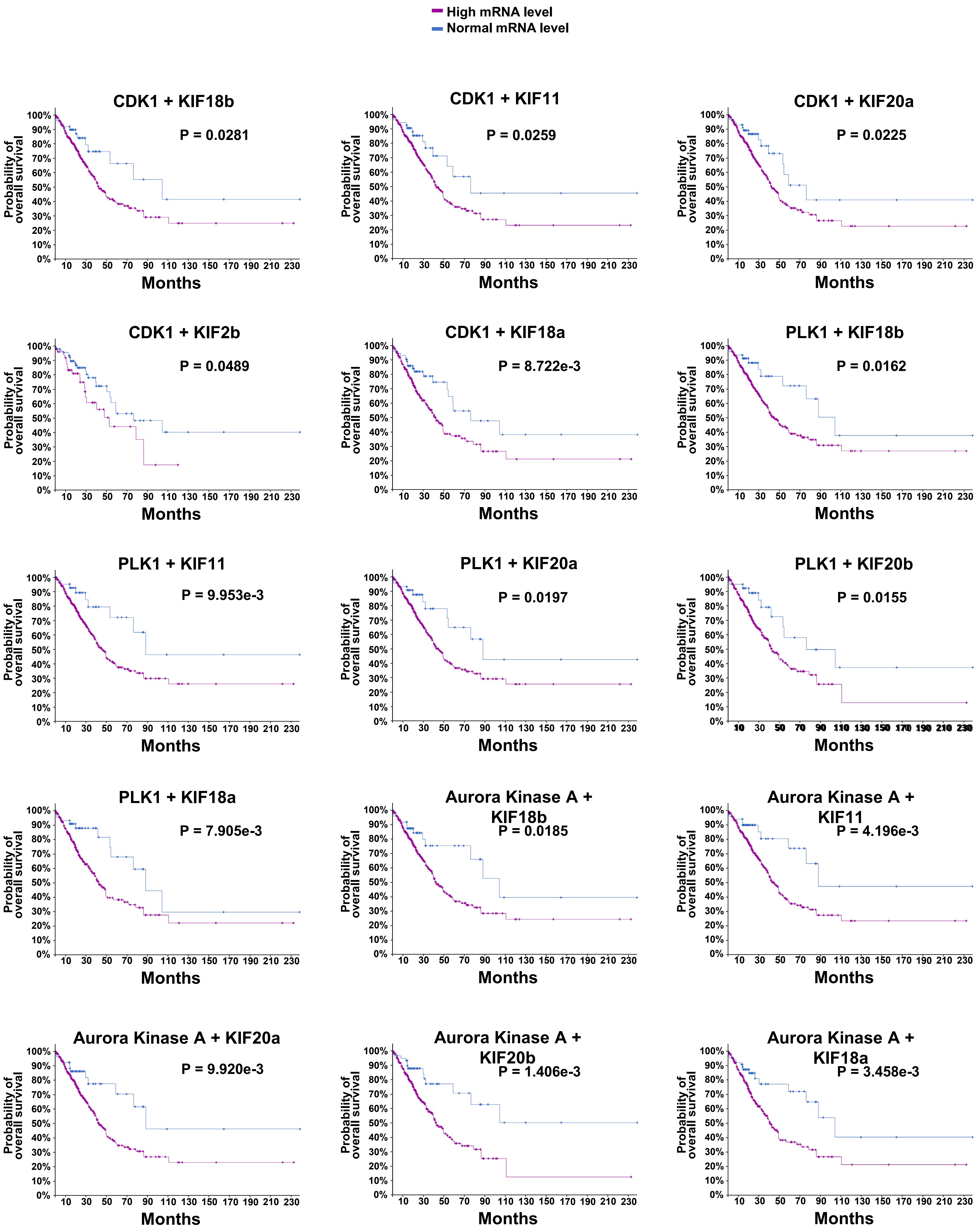

Supplementary Fig 5: The interrogation of differentially expressed genes (Cdk1, Plk1, Aurora Kinase A, KIFC1, KIF2C and KIF22) in polyploid versus diploid cancer cells increased after CYC065 but not vehicle treatments in the lung adenocarcinoma cohort (566 patients) in PanCancer Atlas within The Cancer Genome Atlas (TCGA). (**A**) The consequence of over-expression of each Cyclin B1, Cyclin A2, CDC20, CDC25C, KIF18b, KIF11, KIF20a, KIF20b, Aurora kinase B, and KIF18a species was significantly associated with a high aneuploidy score. (**B**) CDK1 or PLK1 expression was significantly associated with KIF18b, KIF11, KIF20a, KIF20b, Aurora kinase B, or KIF18a expression profiles. (**C**) Over-expression of Cyclin B1, Cyclin A2, CDC20, CDC5C, KIF18b, KIF11, KIF20a, KIF20b, Aurora kinase B, or KIF18a was associated with an unfavorable overall survival in the examined lung cancer cases. (**D**) The combined over-expression of CDK1 pathway (CDK1, PLK1 or Aurora kinase A) members with KIF18b, KIF11, KIF20a, KIF20b or KIF18a expression profiles was associated with an unfavorable overall survival in this lung cancer case cohort.

Supplementary Fig 6

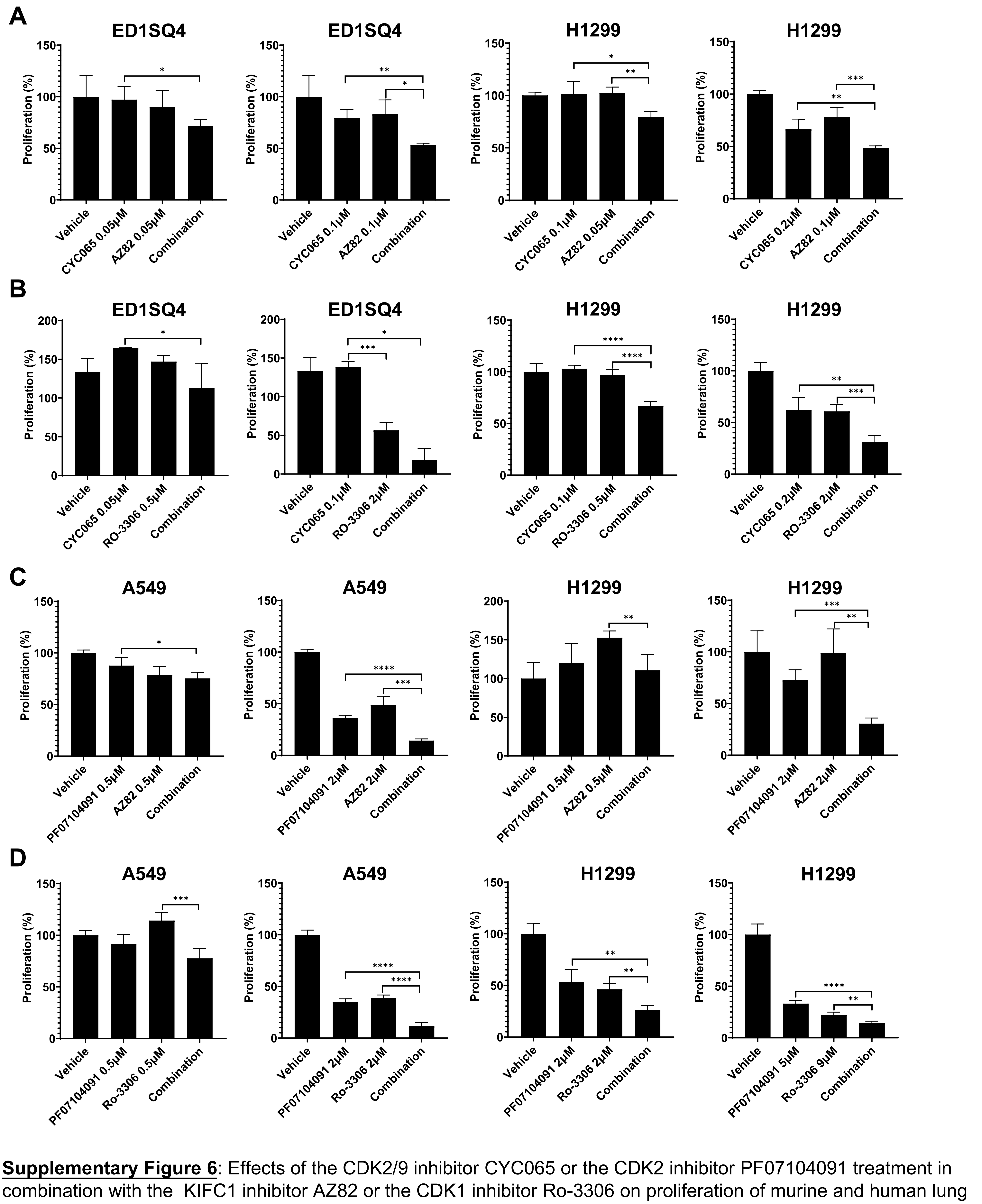

<u>Supplementary Figure 6</u>: Effects of the CDK2/9 inhibitor CYC065 or the CDK2 inhibitor PF07104091 treatment in combination with the KIFC1 inhibitor AZ82 or the CDK1 inhibitor Ro-3306 on proliferation of murine and human lung cancer cell lines. (**A**) Independent CYC065 treatment effects in combination with AZ82 treatment of murine ED1SQ4 and human H1299 lung cancer cell lines. (**B**) Independent CYC065 treatment effects in combination with Ro-3306 in murine ED1SQ4 and human H1299 lung cancer cell lines. (**C**) Independent PF07104091 treatment effects in combination with AZ82 treatment in human A549 and H1299 lung cancer cell lines. (**D**) Independent PF07104091 treatment effects in combination with R0-3306 in human A549 and H1299 lung cancer cells lines. These combination regimens statistically-significantly increased repression of growth in the displayed lung cancer cell lines, as compared to monotherapy. Proliferation assays at Day 4 are shown. Error bars represent standard deviations with the symbols indicating * P < 0.05, ** P < 0.01, *** P < 0.001 and **** P < 0.0001, respectively.

Supplementary Fig 7

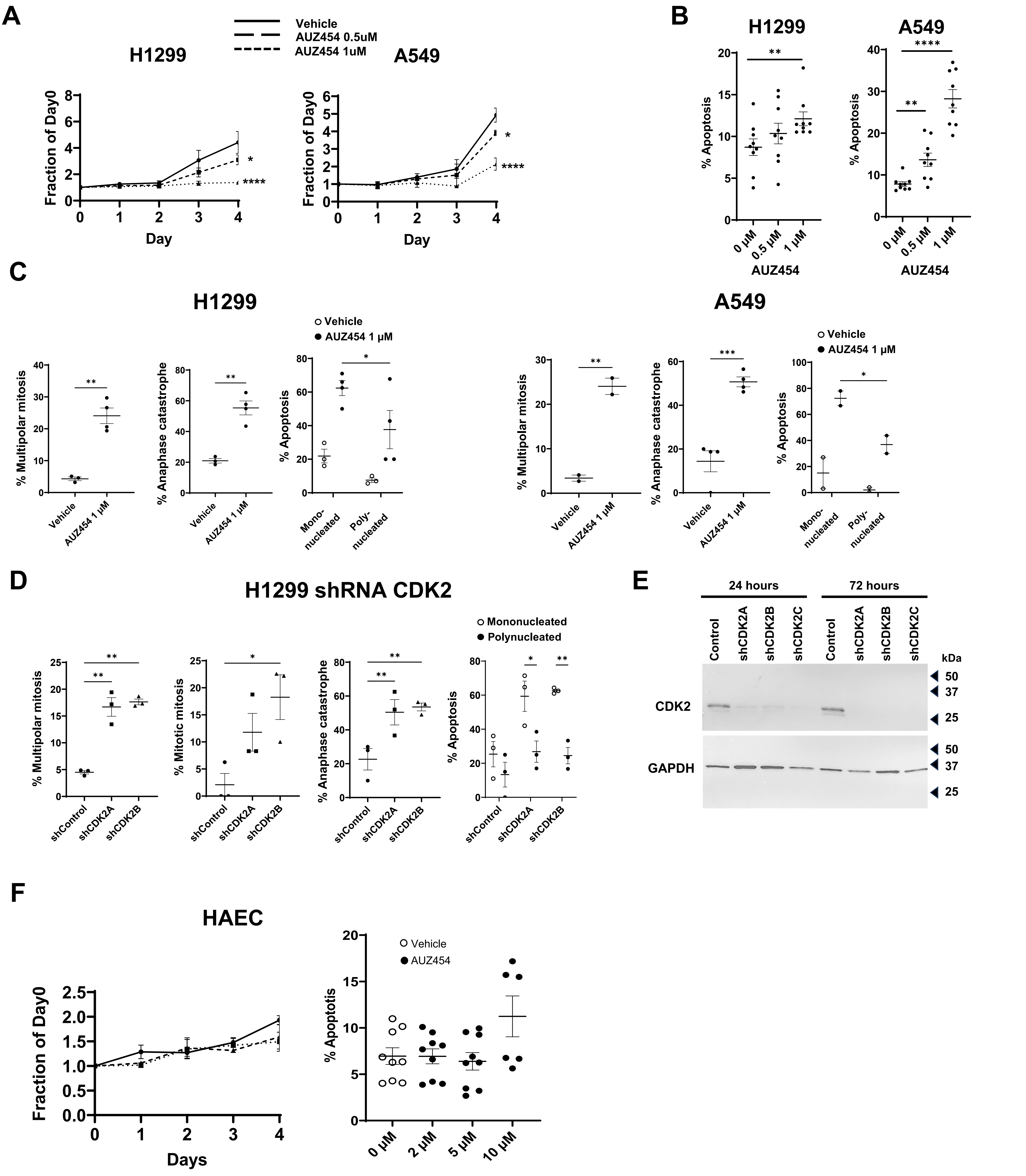

Supplementary Figure 7: Effects of the CDK2 specific inhibitor AUZ454 on proliferation, apoptosis, multipolar mitosis and anaphase catastrophe in lung cancer cells and primary human alveolar epithelial cells (HAEC). (**A**) AUZ454 treatment led to growth inhibition and (**B**) AUZ454 treatment increased apoptosis in a dose-response manner in H1299 and A549 cells. (**C**) AUZ454 treatment augmented multipolar mitosis and anaphase catastrophe in H1299 and A549 cells. (**D**) The shRNA knockdown of CDK2 induced multipolar mitosis, mitotic catastrophe, and anaphase catastrophe in H1299 cells. **(E)** The shRNA knock of CDK2 was validated by immunoblot in H1299 cells. (**F**) AUZ454 treatment did not statistically significantly affect proliferation or augment apoptosis in HAEC. Error bars represent standard deviations with the symbols indicating * $P < 0.05$, ** $P < 0.01$, *** $P < 0.001$ and **** $P < 0.0001$, respectively.

Supplementary Fig 8

A

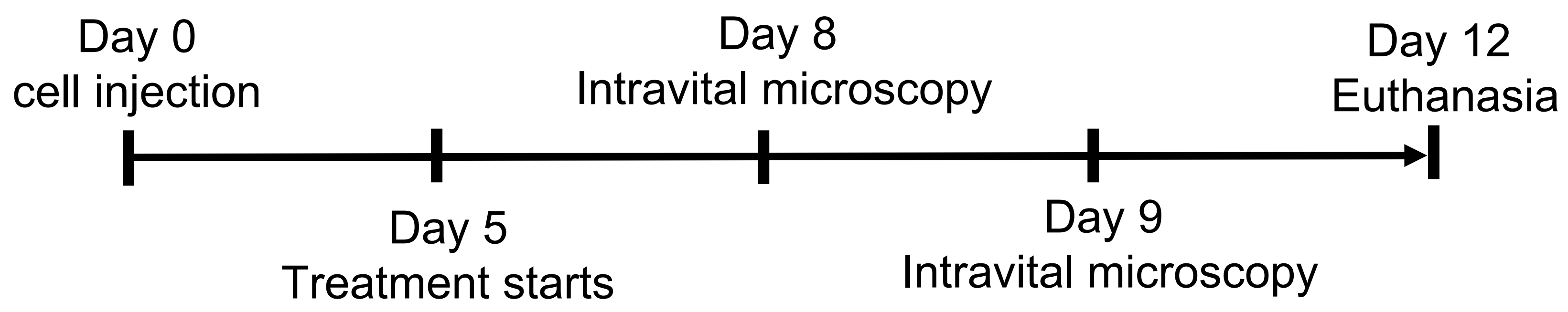

B

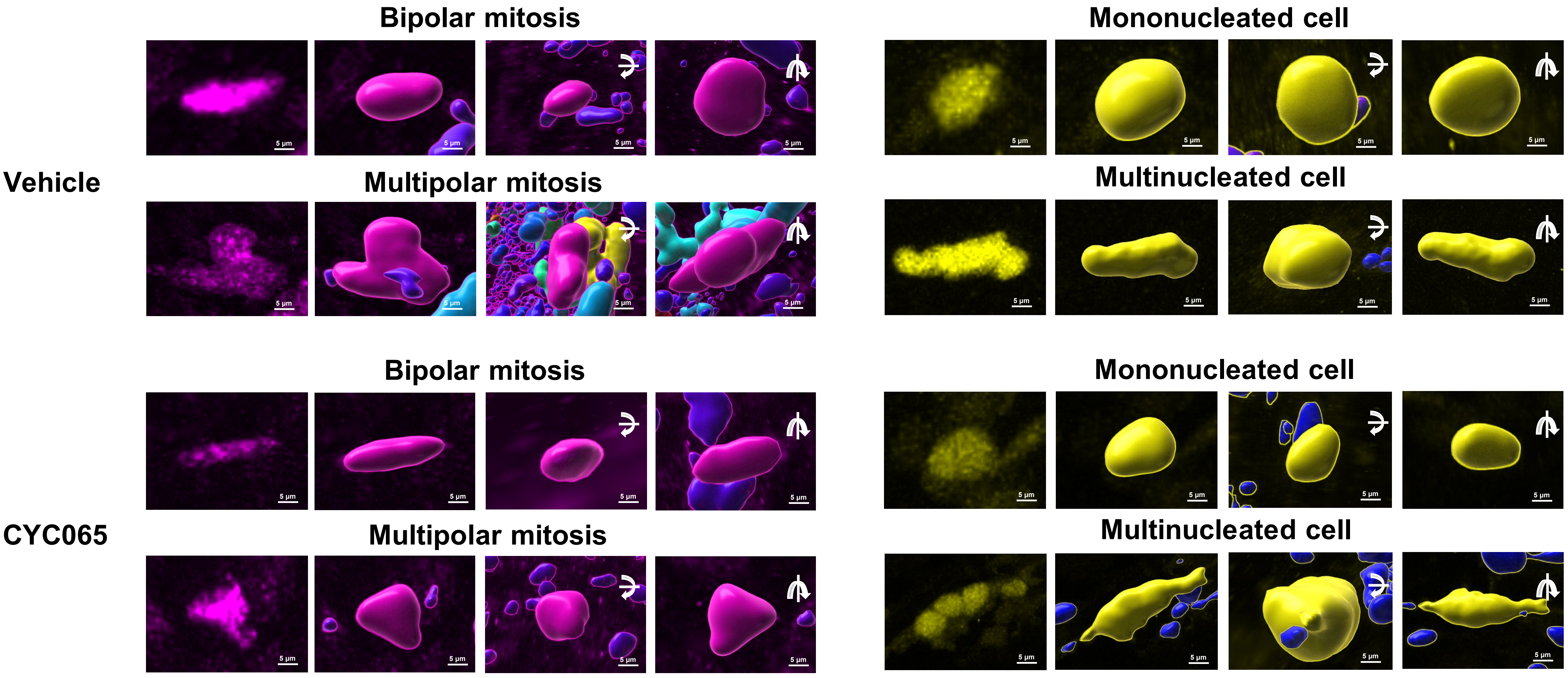

Supplementary Figure 8: Intravital microscopy detection of lung cancer cells in murine ED1SQ4 lung cancers transplanted into syngeneic mice. (**A**) Study schema of treatment and image acquisition are shown. (**B**) Representative fluorescent images and 3D models of bipolar mitotic multipolar mitotic, mononucleated, and multinucleated cellular events from each of the vehicle or CYC065 treatment arms are displayed.
