# Supplementary material for: CDK2 inhibition produces a persistent population of polyploid cancer cells
Source: JCI Insight. 2025 Apr 15;10(10):e189901. doi: 10.1172/jci.insight.189901 (PMC12128980; doi:10.1172/jci.insight.189901)
Supplement: Unedited blot and gel images [file jciinsight-10-189901-s206.pdf]

Supplementary Fig 7e
unedited images

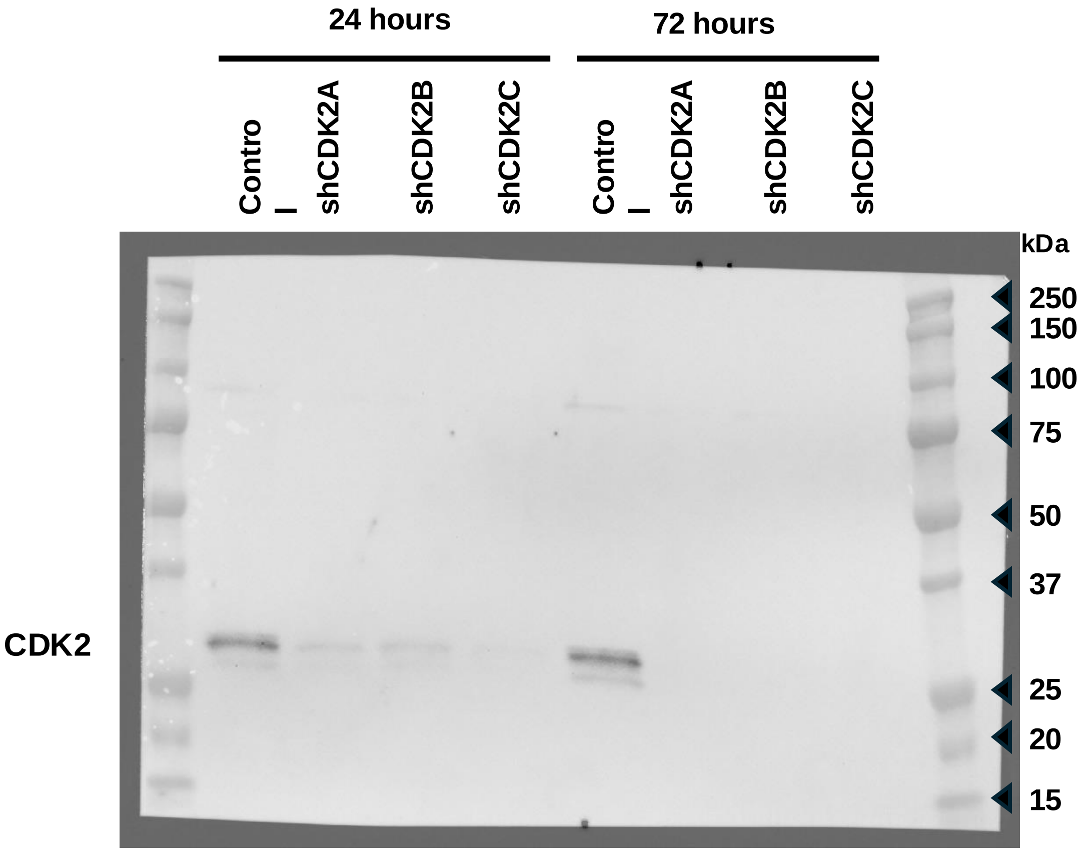

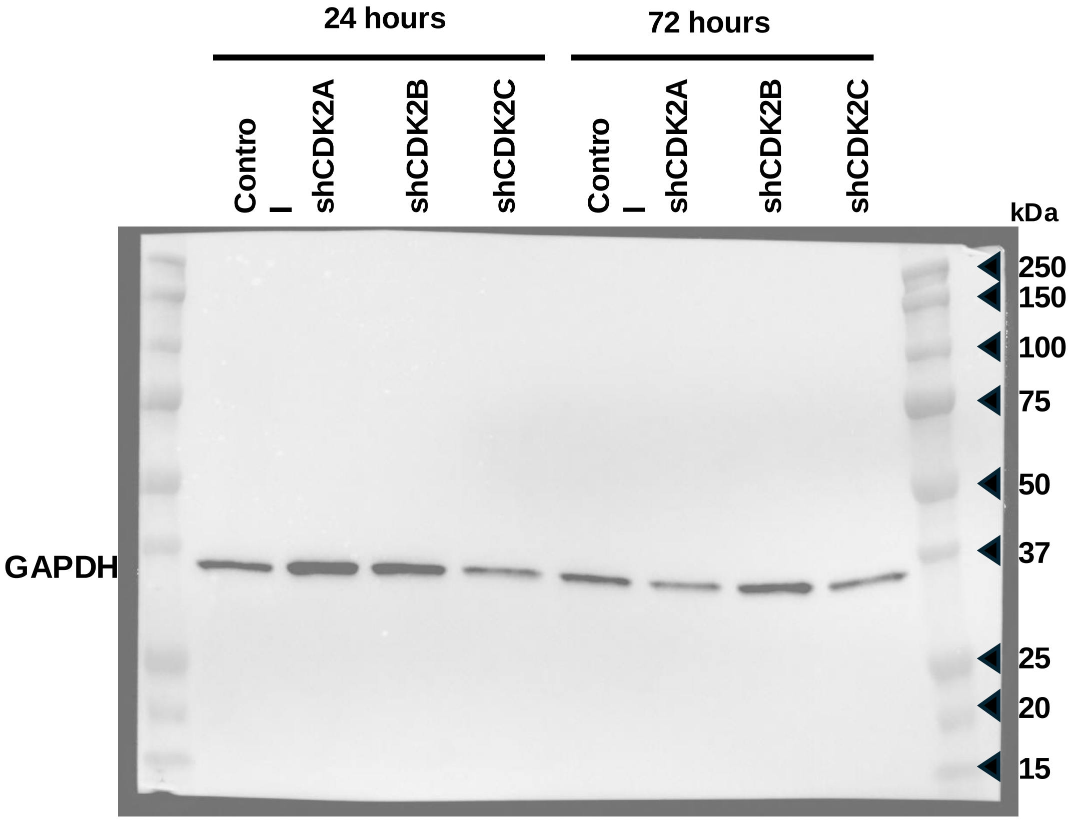

Primary antibody: CDK2 (#ab32147, Abcam)
Second antibody: goat anti-rabbit IgG (#1706516, Bio-RAD)

Primary antibodies: GAPDH (#14C10, Cell Signaling Technology)
Second antibody: goat anti-rabbit IgG (#1706516, Bio-RAD)
